# Paxillin and Hic-5 Interaction with Vinculin Is Differentially Regulated by Rac1 and RhoA

**DOI:** 10.1371/journal.pone.0037990

**Published:** 2012-05-22

**Authors:** Nicholas O. Deakin, Christoph Ballestrem, Christopher E. Turner

**Affiliations:** 1 Department of Cell and Developmental Biology, State University of New York Upstate Medical University, Syracuse, New York, United States of America; 2 Wellcome Trust Centre for Cell-Matrix Research, University of Manchester, Manchester, United Kingdom; King's College London, United Kingdom

## Abstract

Cell migration is of paramount importance to organism development and maintenance as well as multiple pathological processes, including cancer metastasis. The RhoGTPases Rac1 and RhoA are indispensable for cell migration as they regulate cell protrusion, cell-extracellular matrix (ECM) interactions and force transduction. However, the consequences of their activity at a molecular level within the cell remain undetermined. Using a combination of FRET, FRAP and biochemical analyses we show that the interactions between the focal adhesion proteins vinculin and paxillin, as well as the closely related family member Hic-5 are spatially and reciprocally regulated by the activity of Rac1 and RhoA. Vinculin in its active conformation interacts with either paxillin or Hic-5 in adhesions in response to Rac1 and RhoA activation respectively, while inactive vinculin interacts with paxillin in the membrane following Rac1 inhibition. Additionally, Rac1 specifically regulates the dynamics of paxillin as well as its binding partner and F-actin interacting protein actopaxin (α-parvin) in adhesions. Furthermore, FRET analysis of protein:protein interactions within cell adhesions formed in 3D matrices revealed that, in contrast to 2D systems vinculin interacts preferentially with Hic-5. This study provides new insight into the complexity of cell-ECM adhesions in both 2D and 3D matrices by providing the first description of RhoGTPase-coordinated protein:protein interactions in a cellular microenvironment. These data identify discrete roles for paxillin and Hic-5 in Rac1 and RhoA-dependent cell adhesion formation and maturation; processes essential for productive cell migration.

## Introduction

Cell migration is critical for normal development and wound repair, as well as pathophysiologic events such as cancer metastasis. The integrin family of heterodimeric transmembrane proteins are essential for cell migration as they serve as both the physical link between the cell and its microenvironment as well as a conduit for the transmission of force and bidirectional signals necessary for coordination of the cell motility machinery [1].

Integrins cluster in the membrane following their activation by either intracellular or external cues and recruit a functionally diverse array of intracellular proteins to form characteristic adhesion contacts [2]. The formation and stabilization of integrin-mediated adhesions is dependent on the activity of the RhoGTPase family of proteins, in particular Rac1 and RhoA. The activation of Rac1 at the cell periphery stimulates adhesion formation as well as actin-mediated cell protrusion, while RhoA activation promotes adhesion contact maturation and growth as well as cell contractility [3].

Proteins that localize to adhesion contacts can be subdivided into functional groups including but not limited to structural, adaptor and signaling proteins [4]. Paxillin and its closely related family member Hic-5 (ARA55, TGFB1l1) are two such adaptor proteins, which function as molecular scaffolds to spatiotemporally integrate the function and enzymatic activity of a variety of proteins at integrin adhesion sites [5], [6]. In contrast, vinculin performs a structural role and is necessary for adhesion strengthening and force transmission through its interaction with talin and the actin cytoskeleton [7], [8], [9], [10].

**Figure 1:**
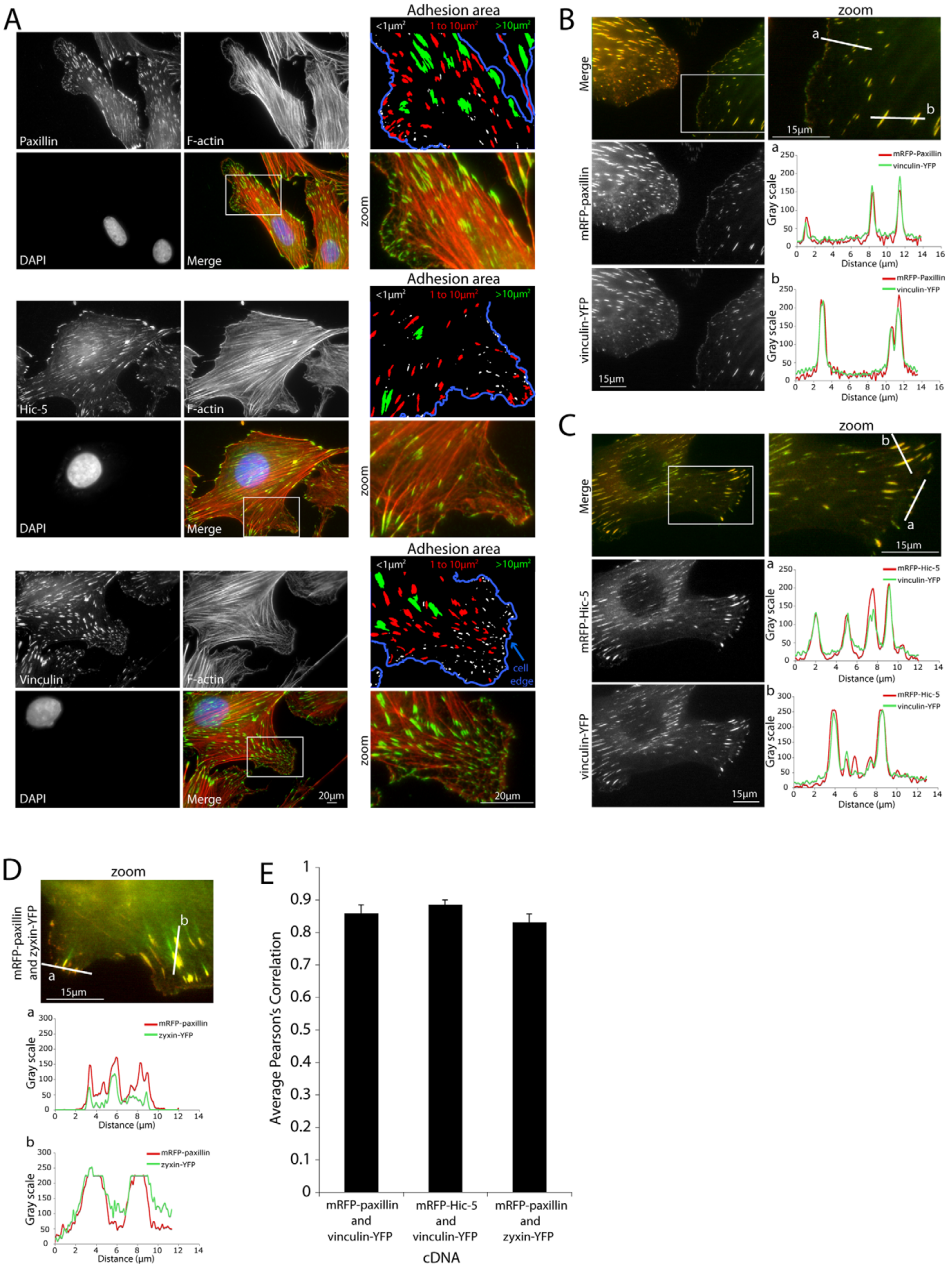
Endogenous and exogenous paxillin, Hic-5 and vinculin colocalize in integrin-mediated adhesions of varying size. (A) Immunofluorescence images of NIH 3T3 cells spread on 2D fibronectin for 4hrs and stained for paxillin (top panels), Hic-5 (middle panels) and vinculin (bottom panels). Masks indicate that the endogenous proteins localize to all adhesions (<1 μm^2^ to >10 μm^2^). Blue line indicates the cell edge as determined by F-actin staining. Representative images of cells co-expressing (B) mRFP-paxillin and (C) mRFP-Hic-5 with vinculin-YFP. Line profiles indicate fluorophore-tagged protein colocalization in all adhesion structures. (D) Representative image and line profiles of mRFP-paxillin and zyxin-YFP colocalization in adhesions. (E) Average Pearson's Correlation analyses indicate a high level of colocalization of exogenous proteins. Error bars are standard errors of the mean.

Paxillin binds directly to vinculin [11] and both proteins are amongst the first recognized members of the integrin adhesome [4], [12], [13]. More recently, Hic-5 was also found to interact with vinculin *in vitro* [14]. Whether the paxillin and Hic-5 interactions with vinculin are functionally and spatially distinct in a cellular environment remains to be determined.

Integrin-extracellular matrix (ECM) adhesion contacts formed on 2D substrates can be classified by their size and location in the cell. Focal complexes are small (<1 µm^2^) Rac1-mediated adhesion contacts that form at the cell periphery, particularly at areas of membrane/lamellipodial protrusion [15], [16], [17], [18]. Focal complexes either disassemble rapidly or transition to the larger (>1 µm^2^) spatiotemporally distinct RhoA- and force-dependent, focal adhesions [19], [20], [21]. Importantly, despite the well-characterized roles and absolute requirement for the RhoGTPases in integrin-mediated adhesion to the ECM [17], [19], [20], [21], [22], [23], the consequences of their activation at a molecular interaction level in a cellular environment and thus how they are able to regulate cell migration is relatively unexplored.

Paxillin has been shown to modulate cell migration through coordinating 2D adhesion disassembly [24] as well as through regulating the activity of the RhoGTPases [5]. Hic-5 has also emerged as a regulator of the RhoGTPases to control cell migration [25], [26] and may also serve a mechanosensory role [27]. Vinculin, in addition to controlling adhesion strengthening, also modulates adhesion turnover [28] to regulate cell migration. More recently paxillin, Hic-5 and vinculin have all been identified as components of adhesion contacts formed during migration through *in vivo*-relevant 3D ECM environments [25], [29]. Strikingly, in contrast to 2D systems, Hic-5 is required for 3D adhesion formation in MDA-MB-231 breast cancer cells, while paxillin regulates 3D adhesion assembly, stabilization and disassembly to control mesenchymal cell invasion and plasticity [25].

To characterize the molecular interactions occurring in adhesion contacts through modulation of discrete RhoGTPase signaling we have used a combination of acceptor photobleaching Fluorescence Resonance Energy Transfer (apFRET), Fluorescence Recovery After Photobleaching (FRAP) as well as fluorescence colocalization and biochemical analyses. Importantly, we provide the first description of RhoGTPase-regulated protein:protein interactions in a cellular context in both 2D and 3D microenvironments. Herein we show that the interaction between paxillin and vinculin in and around adhesions is spatially regulated and is dependent on the activity of Rac1 as well as the activation status of vinculin. In contrast, RhoA activation promotes the interaction of Hic-5 with ‘active’ vinculin in adhesions. We also show that in contrast to 2D adhesions the interaction between Hic-5 and vinculin predominates in 3D adhesions. Additionally, we describe a novel role for Rac1 in regulating paxillin dynamics in adhesions, potentially through promoting an interaction between paxillin and the F-actin binding protein actopaxin (α-parvin) [30].

**Figure 2:**
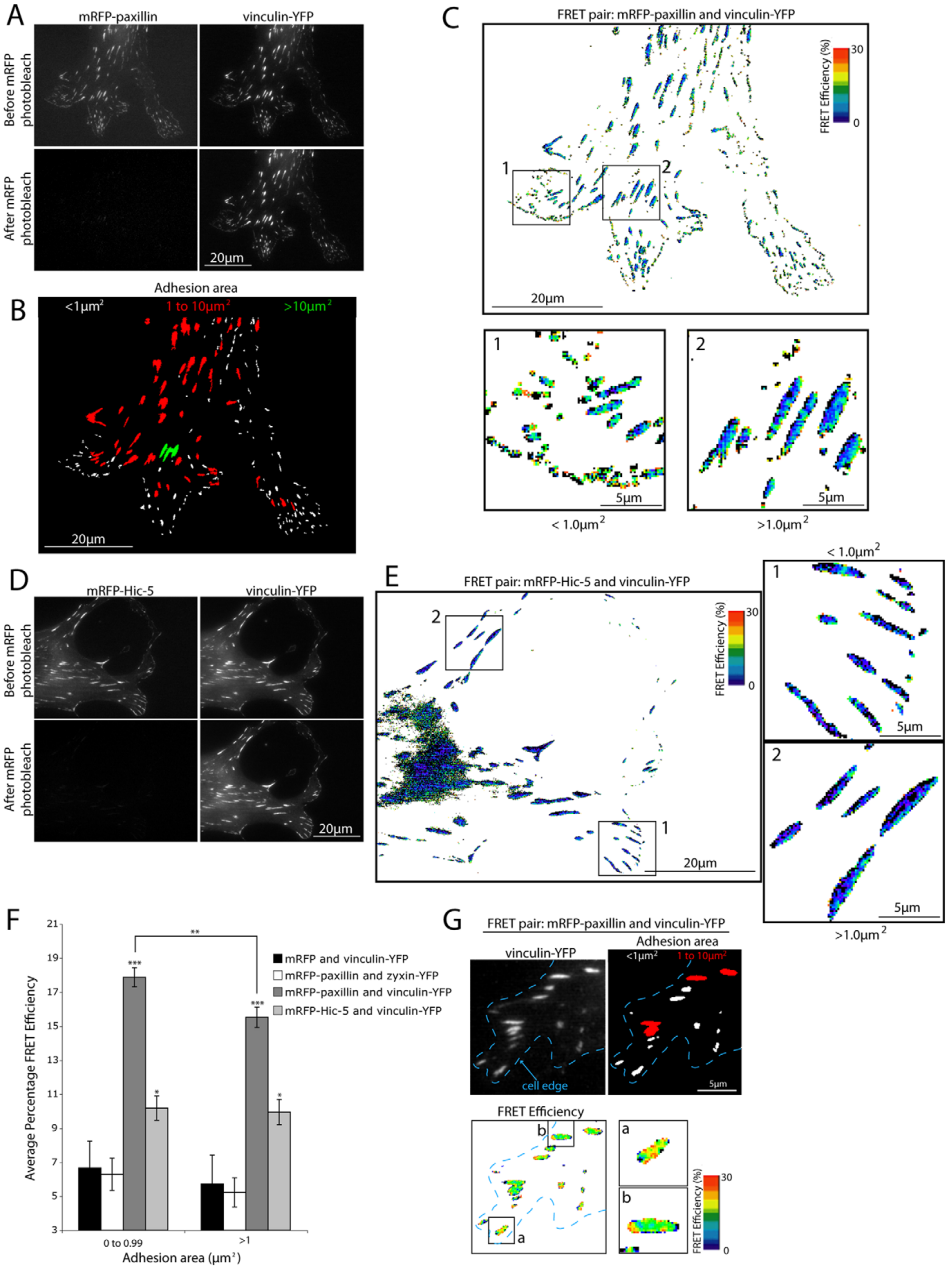
Paxillin and Hic-5 are within FRET proximity of vinculin in adhesions. (A) Raw images of NIH 3T3 cells expressing the donor (vinculin-YFP) and acceptor (mRFP-paxillin) pair used for FRET calculations before and after acceptor photobleaching. The lower panel images highlight the complete photobleaching of the acceptor protein. (B) Mask image of the vinculin-YFP image indicating the variation of adhesion contact area. (C) FRET efficiency image of mRFP-paxillin and vinculin-YFP with zoomed images, indicating FRET in both small and large adhesions. (D) Raw images of NIH 3T3 cells expressing the donor (vinculin-YFP) and acceptor (mRFP-Hic-5) pair used for FRET calculations. (E) FRET efficiency image of mRFP-Hic-5 and vinculin-YFP with zoomed images indicating FRET in both small and large adhesions. (F) Quantitation of average percentage FRET efficiency of indicated FRET pairs in adhesions with small, focal complex-like areas (<1 μm^2^) and larger more mature adhesions (>1 μm^2^). Error bars are standard errors of the mean and are compiled from analysis of all adhesions from a minimum of 9 cells from 3 individual experiments (measurements were made from 450 to 2500 individual adhesions). Statistical analyses are relative to the mRFP and vinculin-YFP FRET control unless otherwise indicated, *  = p<0.05 and *** = p<0.0005. (G) Representative image of a vinculin-YFP-expressing NIH 3T3 cell with adhesions of (a) <1 μm^2^ and (b) 1 to 10 μm^2^ indicating an increase in measured FRET efficiency between mRFP-paxillin and vinculin-YFP in the smaller focal complex-like adhesions. Dashed blue line indicates the cell edge.

## Results and Discussion

### Paxillin and Hic-5 colocalize and interact with vinculin in adhesion contacts

Immunofluorescence imaging of endogenous paxillin and Hic-5, using family member-specific antibodies [25], [26], as well as vinculin in NIH 3T3 fibroblast cells revealed their localization to cell-ECM adhesions of varying size, from small peripheral focal complexes (<1 µm^2^) to larger more mature focal adhesions (>1 µm^2^), as well as larger structures that likely represent merged or spatially inseparable adhesions (>10 µm^2^) (Figure 1A). This subcellular distribution of paxillin and vinculin is consistent with previous studies indicating that these proteins are two of the earliest proteins recruited to assembling integrin-mediated adhesions [18], [31], [32]. A similar analysis of the distribution of fluorophore-tagged proteins, which have been previously shown to function as their wild type counterparts [25], [26], [33], [34], [35], [36], [37] also revealed colocalization between both paxillin and vinculin (Figure 1B) and Hic-5 and vinculin (Figure 1C) in adhesions of all sizes, which was confirmed by Pearson's Correlation coefficient analysis (Figure 1E). Comparable colocalization in all adhesions was also observed between paxillin and zyxin (Figure 1D and E), which are not thought to interact directly.

**Figure 3:**
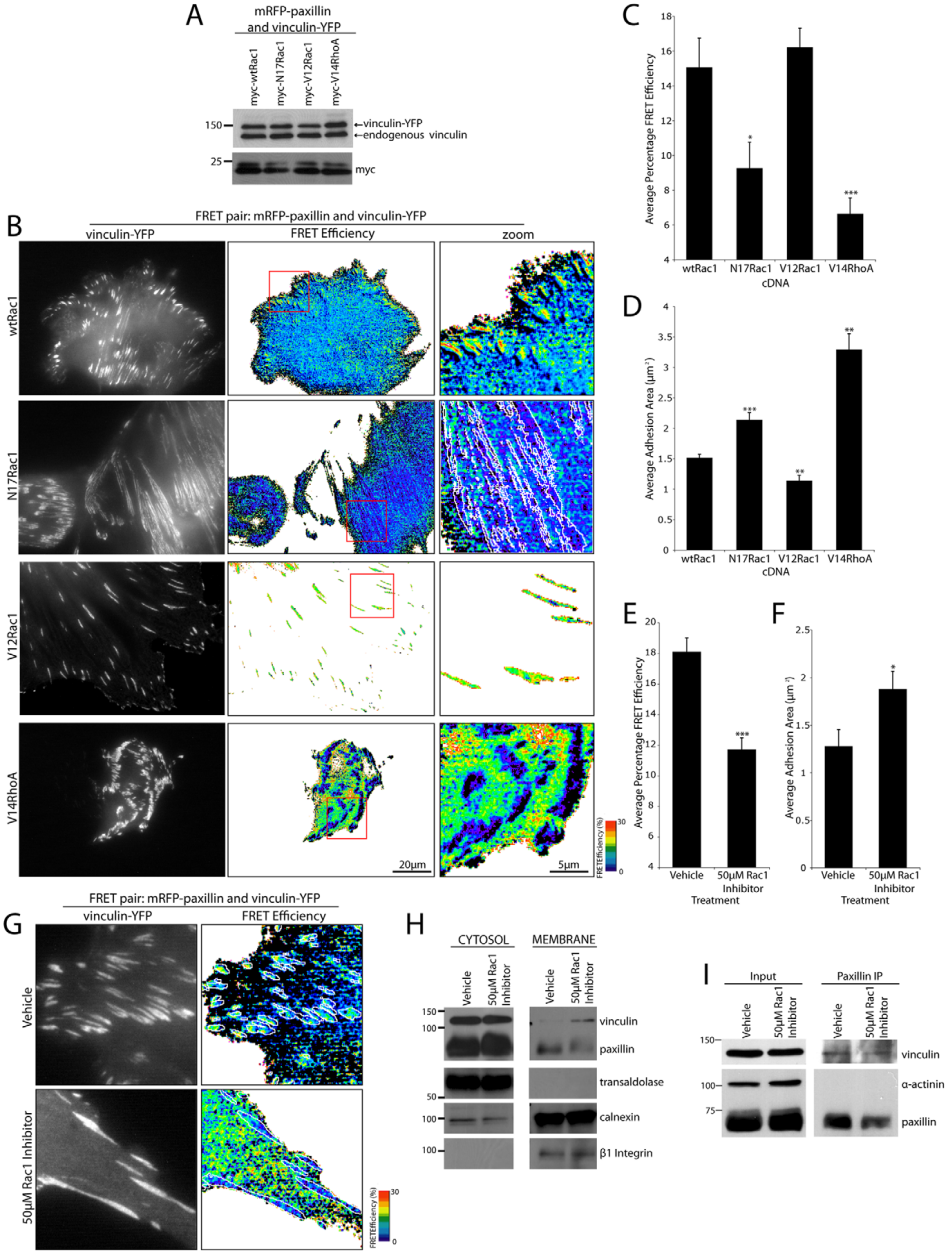
The interaction between paxillin and vinculin in adhesions is spatially regulated by the activity of the RhoGTPases. (A) Western blot of vinculin-YFP and myc-tagged Rac1 and RhoA mutant expression in NIH 3T3 cells used for FRET analyses. (B) Representative donor (vinculin-YFP) and FRET efficiency images indicating that in the presence of wild type (wt) or active (V12Rac1) Rac1, paxillin and vinculin are within FRET proximity. In contrast, inhibition of Rac1 (N17Rac1) or activation of RhoA (V14RhoA) results in a loss of adhesion-localized FRET. FRET image zooms highlight representative FRET pattern of cells as indicated. (C) Quantitation of average FRET efficiency of mRFP-paxillin and vinculin-YFP in all adhesions of cells expressing activation mutants of Rac1 and RhoA. (D) Adhesion area calculations for cells expressing mRFP-paxillin and vinculin-YFP with indicated Rac1 and RhoA mutants. Expression of the active V12Rac1 mutant promotes the formation of smaller adhesions, while the dominant negative N17Rac1 or active V14RhoA constructs induce an increase in adhesion area. Error bars are standard error of the mean from cells used for FRET calculations. (E) Quantitation of average FRET efficiency of mRFP-paxillin and vinculin-YFP in all adhesions of cells in the presence of vehicle (dH_2_0) or 50 μM Rac1 inhibitor (NSC23766). (F) Quantitation of average adhesion area in cells treated with vehicle or 50 μM Rac1 inhibitor. Error bars are standard error of the mean and values calculated from all adhesions from a minimum of 10 cells from 3 individual experiments. * = p<0.05, ** = p<0.005 and *** = p<0.0005. (G) Representative images of NIH 3T3 cells displaying increased vinculin-YFP cytosol/membrane localization indicating positive FRET in both adhesion contacts and areas outside integrin-mediated adhesions. Treatment with 50 μM Rac1 inhibitor decreased FRET in adhesion contacts with cytosolic/membrane FRET still observed. White lines highlight vinculin-YFP-positive adhesion areas. (H) Western blots indicating an increase in endogenous vinculin recruitment to the membrane-enriched fraction of cells treated with 50 μM Rac1 inhibitor. (I) Representative Western blots indicating no effect of the Rac1 inhibitor on the ability of endogenous paxillin and vinculin to coimmunoprecipitate, n = 3 individual experiments.

Importantly, biochemical evidence of a global interaction between vinculin and either paxillin or Hic-5 is well established [11], [14], [38], [39]. However, their spatiotemporal association in cells has not been determined. To investigate the subcellular interaction of vinculin with paxillin and Hic-5 we employed apFRET [40], [41], [42]. This technique can be used to measure the proximity of two fluorophore-tagged proteins and thus assess protein:protein interactions with high sensitivity on the nanometer scale [43], [44].

NIH 3T3 cells expressing vinculin-YFP (donor) and mRFP-paxillin (acceptor) to endogenous levels (Figure S1A) were imaged and the FRET efficiency determined. Images were acquired in the YFP and mRFP channel before and after >95% mRFP photobleaching (Figure 2A) as determined by relative mRFP mean fluorescence intensity before and after photobleaching in cells spread and forming robust adhesions on a 2D fibronectin substrate (Figure 2B). To control for pixel shift aberrations and edge artifacts due to fluctuations in temperature and focus drift during mRFP ablation, the YFP images take before and after mRFP photobleaching were merged and pixel alignment fidelity assessed and corrected. Only images in which all pixels could be aligned as determined by Pearson's Correlation and line profile analyses were used for subsequent FRET calculations (Figure S2B). Positive FRET results in an increase in donor emission (unquenching) after mRFP photobleaching as indicated by an increase in the fluorescence intensity of vinculin-YFP after mRFP destruction (green line) relative to before (red line) (Figure S1B). FRET and thus a direct interaction [45] between paxillin and vinculin was observed in all adhesion contacts (Figure 2C). To determine FRET efficiency strictly within an adhesion, masks were created of cell adhesion contacts from background subtracted vinculin-YFP images before photobleaching and FRET measurements restricted to those areas. Importantly, the FRET observed was not due to pixel shift aberrations or edge artifacts as indicated by image alignments (Figure S1B). Similarly, in cells expressing vinculin-YFP and mRFP-Hic-5 (Figure 2D and E) to endogenous levels (Figure S2A) a direct interaction between the two proteins was also observed (Figure 2E and Figure S2B). Only low background levels of FRET (approximately 6%; Figure 2F) were observed in control cells expressing vinculin-YFP and mRFP vector (Figure S2C). Importantly, despite the high degree of colocalization between paxillin and zyxin the level of FRET (Figure S2D) was similar to that observed with vinculin-YFP and mRFP (Figure 2F), suggesting that these two proteins do not interact in adhesion contacts. These data indicate that in a cellular environment, vinculin colocalizes and directly interacts with both paxillin and Hic-5 in adhesion contacts.

**Figure 4:**
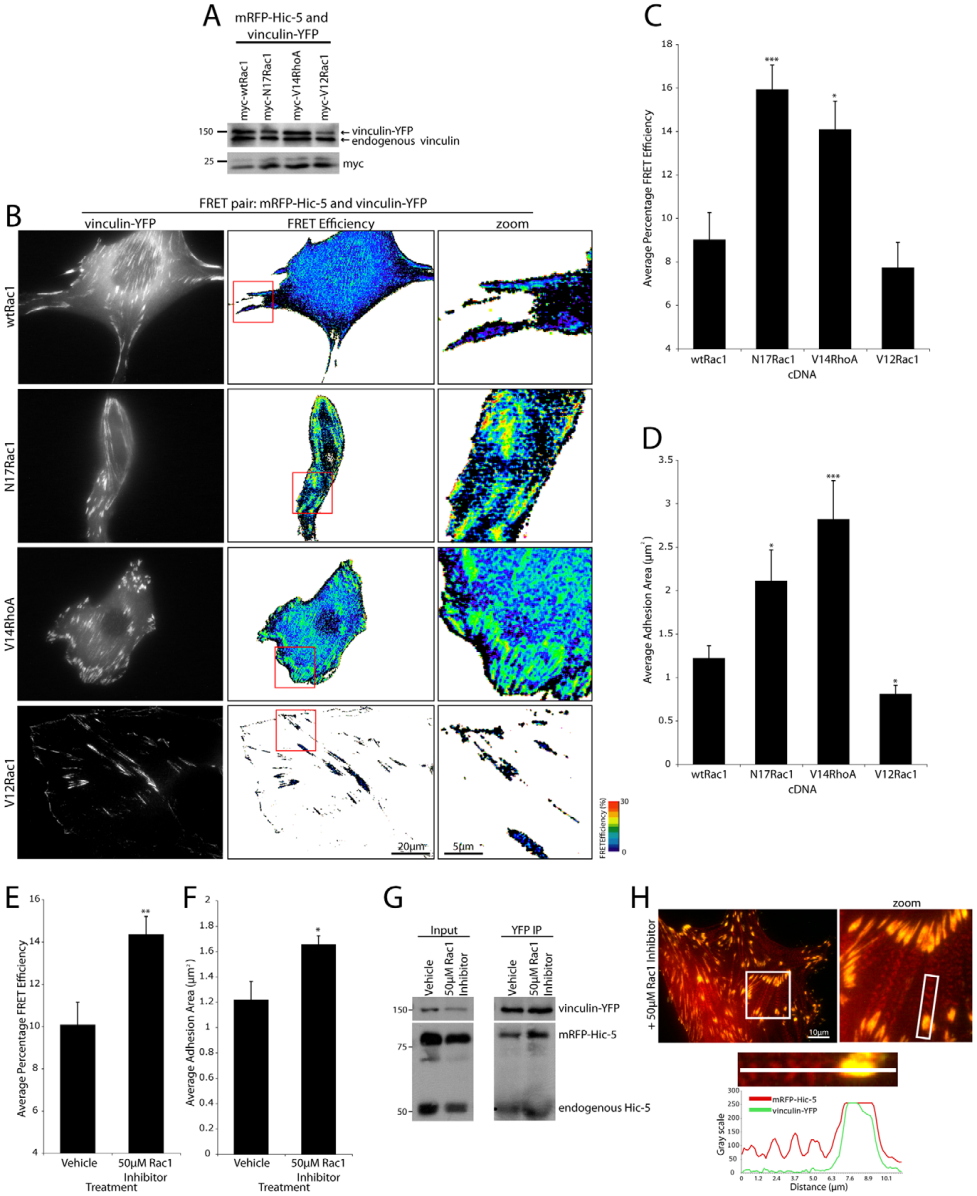
Rac1 inhibition or RhoA activation promotes mRFP-Hic-5 and vinculin-YFP FRET in adhesions. (A) Western blots of donor and myc-tagged Rac1 and RhoA mutant expression. (B) Representative images of mRFP-Hic-5 and vinculin-YFP FRET in adhesions of cells expressing wtRac1, N17Rac1, V14RhoA or V12Rac1 constructs. FRET image zooms highlight representative FRET pattern of cells as indicated. (C) Quantitation of the FRET efficiency between mRFP-Hic-5 and vinculin-YFP in all adhesions and (D) average adhesion area in the presence of Rac1/RhoA activation mutants. Error bars represent standard error of the mean and are calculated from a minimum of 10 cells from 3 individual experiments. (E) Quantitation of the FRET efficiency between mRFP-Hic-5 and vinculin-YFP and (F) average adhesion area in the presence of 50 μM Rac1 inhibitor. Error bars are standard error of the mean from a minimum of 13 cells from 4 individual experiments. * = p<0.05, ** = p<0.005 and *** = p<0.0005. (G) Western blots of vinculin-YFP and Hic-5 coimmunoprecipitation. A small but consistent increase in the interaction of vinculin-YFP with both endogenous and fluorophore-tagged Hic-5 was seen upon Rac1 inhibition. Data is representative of n = 4 individual experiments. (H) Images and line profile of mRFP-Hic-5 localization to stress fibers upon Rac1 inhibition.

### Paxillin exhibits an increased interaction with vinculin in small (<1 µm2) adhesions

The formation and maturation state of adhesion contacts have been shown to be dependent on the activity of different signaling proteins, for example the RhoGTPases [17], [20]. Furthermore, the various types of integrin-mediated adhesion have been reported to have distinct molecular compositions [46], [47], [48]. To assess whether the type or maturation state of the adhesion contact influences the interaction between either paxillin or Hic-5 and vinculin, the FRET efficiency of the respective protein pairs was quantified in adhesions with areas of <1 µm^2^ or >1 µm^2^. Interestingly, there was a highly significant increase in the interaction between paxillin and vinculin in the peripheral small adhesions (focal complexes) relative to the larger adhesions (focal adhesions) (Figure 2F and G). In contrast, no significant difference was observed in the FRET efficiency between Hic-5 and vinculin in adhesions regardless of their size (Figure 2F). These data suggest that the interaction between paxillin and vinculin is spatially regulated and is influenced by, or may even dictate the maturation state of the adhesion contact.

**Figure 5:**
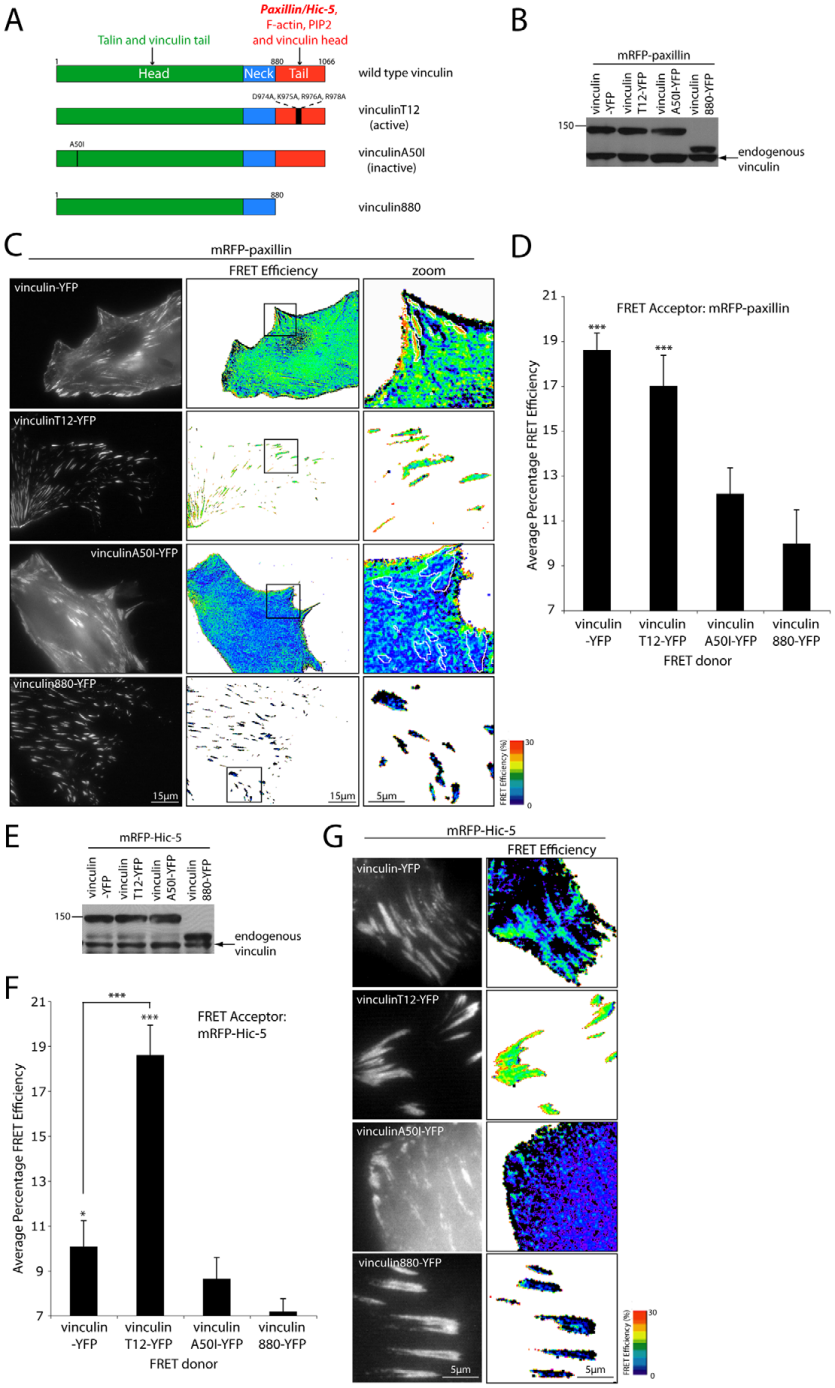
Vinculin activation state spatially regulates its interaction with paxillin and Hic-5. (A) Schematic of vinculin highlighting the distinct domains and associated binding partners as well as mutant constructs used for FRET experiments. (B) Western blot of vinculin-YFP (FRET donor) mutant protein expression in mRFP-paxillin-expressing NIH 3T3 cells. (C) Representative images and (D) quantitation of vinculin-YFP activity mutant's FRET efficiency with mRFP-paxillin. Error bars are standard error of the mean from a minimum of 12 cells from 3 individual experiments. Statistical analyses are relative to the vinculin880-YFP mutant, which lacks the paxillin interacting domain. (E) Western blot of vinculin-YFP (FRET donor) mutant protein expression in mRFP-Hic-5-expressing NIH 3T3 cells. (F) Quantitation and (G) representative images of mRFP-Hic-5 FRET with vinculin-YFP mutants. * = p<0.05 and *** = p<0.0005.

### The activity of the RhoGTPases, Rac1 and RhoA, regulates the spatial localization of the paxillin-vinculin interaction

The assembly of nascent focal complexes and their maturation into focal adhesions is regulated by the activity of Rac1 and RhoA respectively [17], [20]. Furthermore, active Rac1 has been shown to colocalize predominantly with focal complexes at the leading edge of cell protrusions [49], [50], [51]. Therefore, we hypothesized that since paxillin and vinculin exhibit elevated FRET efficiency in focal complexes their interaction may be promoted by the activation of Rac1. To address this possibility, NIH 3T3 cells transfected with mRFP-paxillin and vinculin-YFP were cotransfected with wild type or mutant forms of myc-tagged Rac1 (Figure 3A). Cells expressing dominant negative (N17) Rac1 exhibited a significant decrease in the interaction between paxillin and vinculin in adhesions as indicated by decreased FRET efficiency, while expression of the dominant active (V12) Rac1 resulted in robust paxillin and vinculin interaction in small adhesions throughout the cell (Figure 3B and C). Additionally, N17Rac1 cells displayed a significant increase in average adhesion area (Figure 3D) indicative of a shift towards more RhoA-mediated mature focal adhesions and consistent with previous reports indicating that the activities of Rac1 and RhoA may exhibit a reciprocal relationship [52]. Indeed, analysis of myosin 2 activation, a downstream effector of RhoA, indicated an increase in myosin light chain 2 (MLC2) phosphorylation upon expression of N17Rac1, which is indicative of a shift towards RhoA-mediated adhesion signaling (Figure S3A and B).

**Figure 6:**
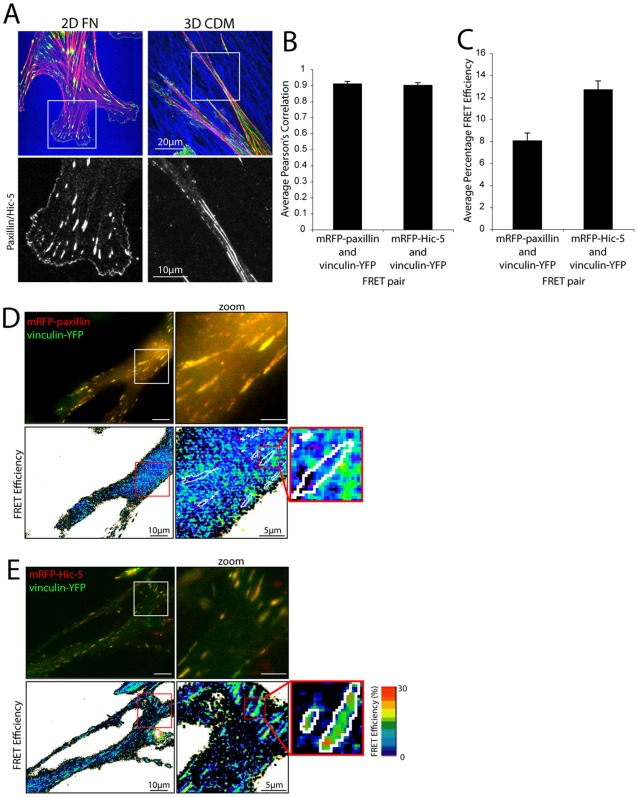
Vinculin preferentially interacts with Hic-5 rather than paxillin in 3D adhesion contacts. (A) Images of NIH 3T3 cells spread on 2D fibronectin (FN) or 3D cell-derived matrix (CDM) for 4hrs stained for paxillin/Hic-5 (green), fibronectin (blue) and F-actin (red). (B) Average Pearson's Correlation analyses of mRFP-paxillin or mRFP-Hic-5 with vinculin-YFP in 3D adhesions reveals no significant difference in colocalization. (C) Quantitation and representative images of (D) mRFP-paxillin and (E) mRFP-Hic-5 FRET efficiency with vinculin-YFP in 3D adhesions demonstrating FRET between vinculin-YFP and mRFP-Hic-5 but not mRFP-paxillin in 3D adhesions. Error bars are standard error of the mean and are calculated from a minimum of 15 cells from 3 individual experiments.

**Figure 7:**
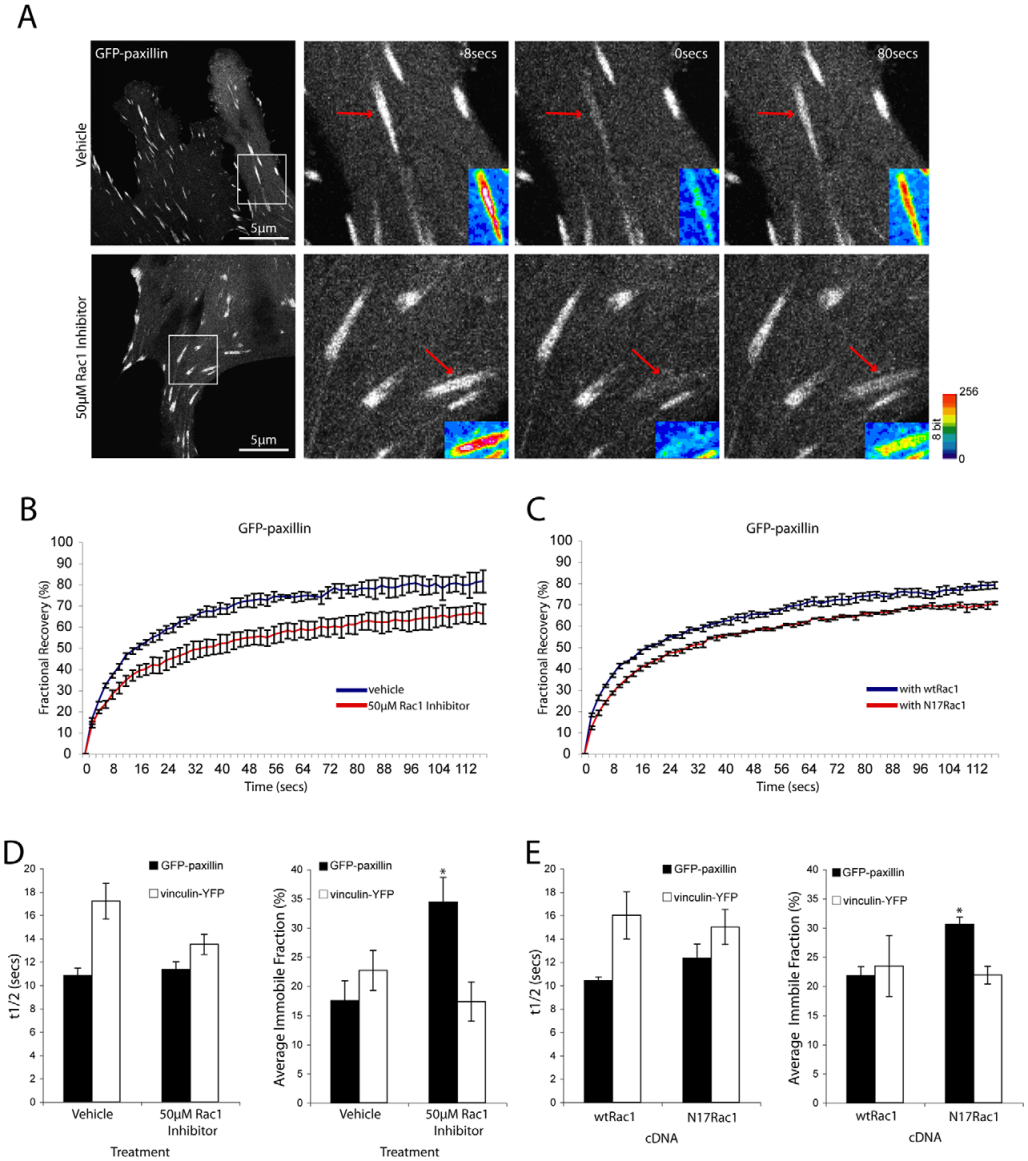
Rac1 inhibition specifically affects the dynamics of paxillin in adhesions. (A) Images of FRAP time series of GFP-paxillin-expressing NIH 3T3 cells ±50 μM Rac1 inhibitor. Inset are pseudo-colored images of the adhesions undergoing FRAP analysis highlighting a reduction in the GFP-paxillin recovery. (B) Compiled fluorescence recovery curves for GFP-paxillin in adhesions from n = 3 individual experiments ± 50μM Rac1 inhibitor and (C) with wtRac1 or N17Rac1. (D) Quantitation of the t1/2 of fluorescence recovery and immobile fraction for GFP-paxillin and vinculin-YFP ±50 μM Rac1inhibitor. (E) Quantitation of the t1/2 of fluorescence recovery and immobile fraction for GFP-paxillin and vinculin-YFP with wtRac1 or N17Rac1. FRAP analyses reveal a significant increase in the immobile fraction of GFP-paxillin, but not vinculin-YFP in cells with decreased Rac1 activity. Error bars are standard error of the mean. * = p<0.05.

To formally test whether RhoA activation may promote the loss of paxillin-vinculin interaction, cells were transfected with the active form of RhoA (V14) (Figure 3A). This also resulted in a significant loss of FRET between paxillin and vinculin in adhesion contacts (Figure 3B and C) and an increase in average adhesion area (Figure 3D) along with an increase in MLC2 phosphorylation (Figure S3A and B). Importantly, the loss of interaction was not due to a decrease in paxillin or vinculin colocalization in adhesions as no change in the Pearson's Correlation coefficient was observed (Figure S4A). The role of Rac1 in regulating the interaction between paxillin and vinculin was further evaluated using the Rac1 inhibitor (NSC23766). Treatment with the Rac1 inhibitor resulted in a significant loss of paxillin and vinculin interaction in adhesions (Figure 3E), increased MLC2 activity (Figure S3C and D) as well as an increase in adhesion area (Figure 3F) without a decrease in their colocalization (Figure S4B). Interestingly, in conjunction with the significant decrease in paxillin and vinculin interaction in adhesions following RhoA activation or Rac1 inhibition, there was a striking and significant concomitant increase in relative FRET efficiency between paxillin and vinculin in the plasma membrane and/or cytosol relative to the FRET efficiency observed in adhesion contacts (Figure 3B, G and Figure S5).

**Figure 8:**
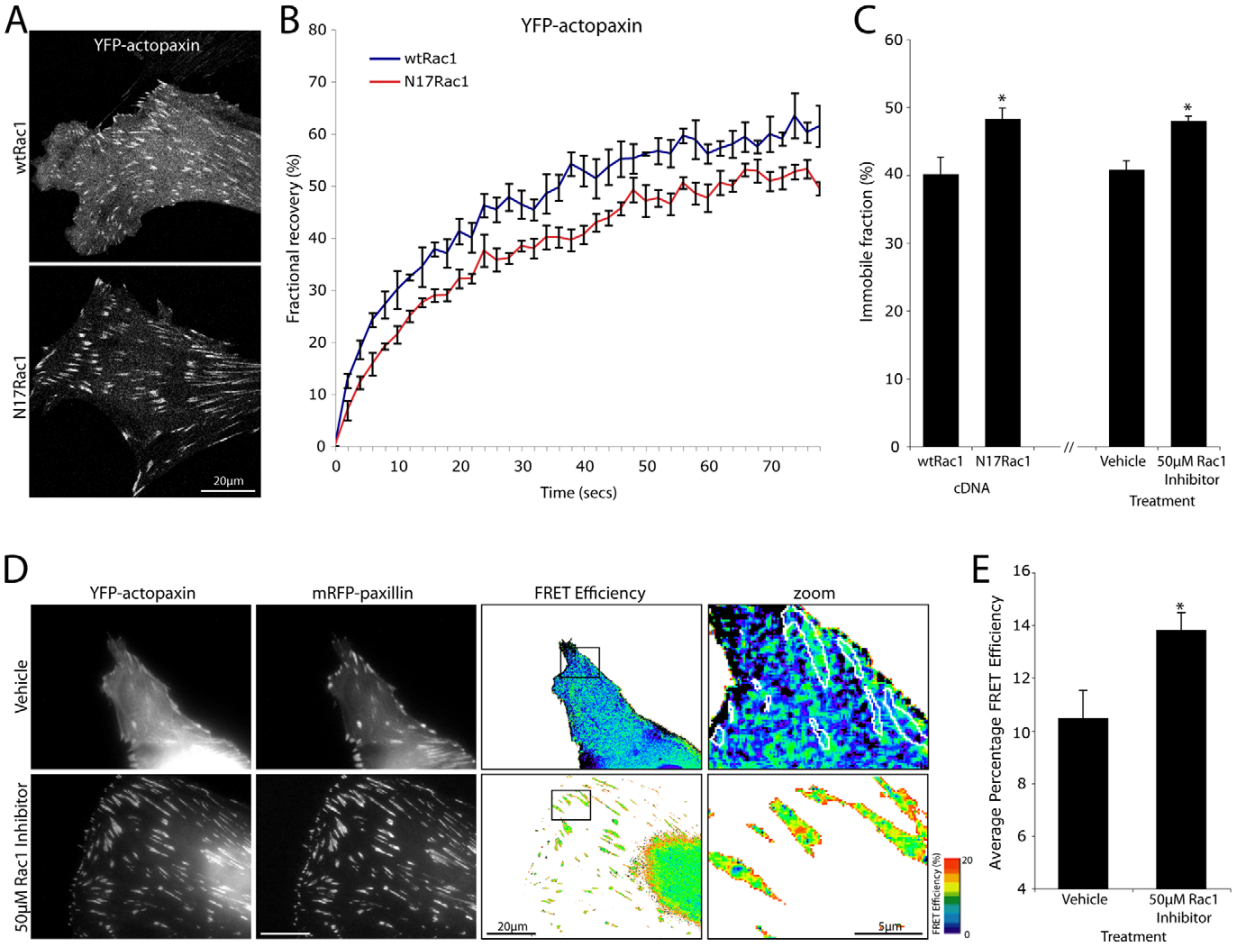
Paxillin is immobilized in adhesions upon Rac1 inhibition through an increased interaction with actopaxin. (A) Representative images of YFP-actopaxin-expressing NIH 3T3 cells used for FRAP analyses. YFP-actopaxin exhibits robust adhesion localization but a reduction in non-adhesion localization upon Rac1 inhibition. (B) Compiled FRAP recovery curves for YFP-actopaxin in the presence of wtRac1 or N17Rac1. Error bars represent standard error of the mean and n = 3 individual experiments. (C) Immobile fraction quantitation for YFP-actopaxin with wtRac1, N17Rac1 and ±50 μM Rac1inhibitor. Error bars are standard error of the mean from a minimum of n = 3 individual experiments. (D) Images and (E) quantitation of YFP-actopaxin and mRFP-paxillin FRET in adhesion contacts upon Rac1 inhibition. Error bars are standard error of the mean and are from all adhesions from a minimum of 11 cells from 3 individual experiments. * = p<0.05.

Vinculin is known to associate with the plasma membrane through its direct interaction with phosphatidylinositol-4,5-bisphosphate (PIP2) [53]. Indeed, along with F-actin and talin binding this interaction is necessary for complete vinculin activation and function [7], [54]. We used cell fractionation to examine whether the FRET observed outside the adhesion contacts was due to increased vinculin recruitment to the cytosol or membrane compartments. No significant difference in either paxillin or vinculin protein levels in the transaldolase-enriched cytosolic fraction could be detected (Figure 3H). In contrast, a significant 2.7 fold (±0.6; p<0.05) increase in endogenous vinculin but no increase in paxillin was observed in the membrane fraction, which was enriched in both calnexin and β1 integrin (Figure 3H).

**Figure 9:**
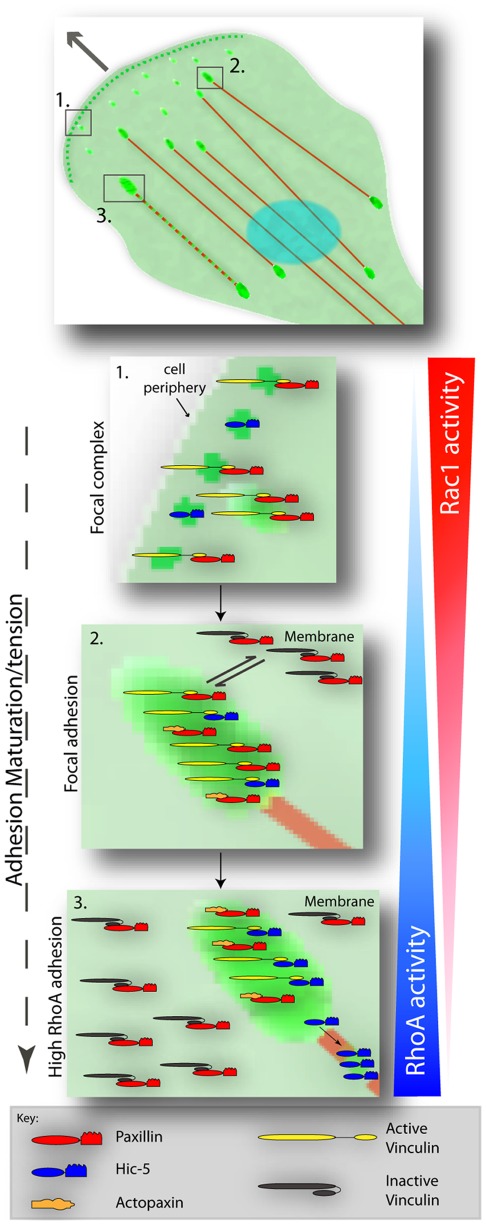
Schematic representation highlighting paxillin and Hic-5 molecular interactions during RhoGTPase-dependent adhesion maturation. (1) At the leading edge of a migrating cell high Rac1 activity stimulates nascent adhesion formation in which the interaction of paxillin with vinculin in its active conformation is stimulated to promote adhesion stabilization and maturation. (2) As adhesion contacts mature the levels of Rac1 and RhoA likely balance and an equilibrium is reached and a steady state maintained whereby paxillin, and to a lesser extent Hic-5, is interacting with active vinculin in adhesions. Furthermore, at steady state paxillin is also in a complex with vinculin in its inactive state in the membrane. (3) Elevated levels of RhoA activity as is observed in cells under tension induces the loss of paxillin-vinculin interaction and a distinct switch to paxillin binding to actopaxin, which likely stabilizes the complex in adhesions. An increase in RhoA and concomitant decrease in Rac1 activity promotes the direct interaction of Hic-5 with active vinculin in contractile adhesions and stimulates the redistribution of Hic-5 to stress fibers to function potentially in adhesion strengthening and/or mechanosensing.

Taken together, these data suggest that Rac1 inhibition and/or RhoA activation may promote the redistribution of vinculin to the plasma membrane and that this pool also directly interacts with paxillin. This observation may also explain why we failed to observe a significant change in the amount of co-precipitating paxillin and vinculin (Figure 3I; p = 0.44, n = 3 individual experiments) despite the loss of FRET between paxillin and vinculin in adhesions. These data are also consistent with the role of RhoA in the activation of PIP 5-kinase, which is essential in the generation of PIP2 [55], [56], and may in turn lead to an increase in vinculin membrane recruitment [57].

### Rac1 inhibition and RhoA activation promotes Hic-5 and vinculin FRET in adhesions and promotes Hic-5 translocation to stress fibers

Hic-5 has been shown to modulate the activity of RhoGTPases in multiple cell types, for example during cancer cell 3D invasion [25] and epithelial to mesenchymal transition (EMT) [26]. Whether Hic-5 activity/function is responsive to RhoGTPase activity is unknown. Interestingly, when we analyzed the impact of manipulating RhoGTPase activity on the Hic-5-vinculin interaction we found that in stark contrast to paxillin (Figure 3), expression of N17Rac1 or dominant active V14RhoA (Figure 4A) resulted in a highly significant increase in Hic-5 and vinculin interaction in adhesion contacts (Figure 4B and C), along with increased adhesion contact area (Figure 4D), with no change in colocalization (Figure S4C). In contrast, expression of dominant active V12Rac1 (Figure 4A) resulted in background levels of FRET between mRFP-Hic-5 and vinculin-YFP (Figure 4B and C) with a significant decrease in average adhesion area observed (Figure 4D). Pharmacologic inhibition of Rac1 produced similar effects to the N17Rac1 and V14RhoA-expressing cells resulting in an increase in Hic-5 and vinculin interaction in adhesions (Figure 4E, F and Figure S4D). Interestingly, unlike paxillin no significant FRET was observed in the membrane between Hic-5 and vinculin (Figure 4B) regardless of GTPase activity. Furthermore, biochemical analysis of Hic-5 and vinculin interaction revealed a small global increase (14.9% increase +/−6.0, p<0.05, n = 4) in coimmunoprecipitating endogenous and fluorophore-tagged Hic-5 with vinculin-YFP upon Rac1 inhibition (Figure 4G). These results suggest that paxillin and Hic-5, although structurally related, may have discrete rather than redundant roles at a protein:protein interaction as well as a functional level [25], [58], [59].

The functional diversity of paxillin and Hic-5 is further highlighted by their distinct cellular localization characteristics. Paxillin is predominantly restricted to all sites of integrin adhesion to the ECM through its recruitment via the C-terminal LIM domains [60], while Hic-5 can translocate from adhesions to stress fibers upon the application of external tensile strain [61], [62]. Consistent with Hic-5 being responsive to mechanical stimuli, Hic-5 exhibited striking redistribution to stress fibers in cells treated with the Rac1 inhibitor (Figure 4H) as well as cells expressing N17Rac1 and V14RhoA (data not shown). Importantly, Hic-5 stress fiber localization was found to be independent of its interaction with vinculin, which did not colocalize or exhibit FRET with Hic-5 in these structures (Figure 4H). Therefore, these data show that Hic-5 responds to RhoA activation-mediated intracellular tension through increased association with vinculin in adhesions as well as its redistribution to stress fibers. These data also indicate that despite extensive sequence conservation between paxillin and Hic-5, their molecular interactions and spatiotemporal localizations can be regulated by the RhoGTPases to promote functional diversity.

### Vinculin “activation” state dictates the spatial interaction with paxillin and Hic-5

Vinculin is comprised of three distinct domains, an amino-terminal globular head, a flexible proline-rich linker and the paxillin/Hic-5 interacting carboxyl-terminal tail [63], [64]. Each domain exhibits the capacity to interact with a variety of proteins including talin [65], PIP2 [66] and F-actin [67] (Figure 5A). Furthermore, a high affinity intramolecular association between the head and tail domain of vinculin is responsible for maintaining vinculin in a ‘closed’ inactive state [64], [65]. Using a conformationally sensitive intramolecular FRET probe, it has been shown that vinculin activation is predominantly restricted to sites of integrin-mediated adhesion. In contrast, inactive vinculin is distributed throughout the membrane/cytosol but was also seen at adhesion contacts [68]. Importantly, the role of vinculin activation on its ability to bind paxillin and/or Hic-5 has not been determined.

In order to test the effect of vinculin activation state on its interactions with paxillin/Hic-5 we performed apFRET using the ‘active’ T12 and ‘inactive’ A50I vinculin mutants (Figure 5A). The vinculinT12 mutant lacks the ability to exhibit the autoinhibitory head-tail interaction [69], while the vinculinA50I mutant has enhanced head-tail affinity and is incapable of interacting with talin but both retain the PIP2-binding site [64], [69]. A vinculin880 mutant previously shown not to interact with paxillin, as it lacks the paxillin and Hic-5 interacting carboxyl-terminus [63], [70], was also used as a negative/background control for subsequent FRET experiments. Western blot analysis revealed that all of the vinculin constructs were expressed to similar levels relative to the endogenous protein (Figure 5B) and consistent with previous studies [70], the vinculin880 and T12 mutants increased average adhesion size, while expression of the A50I mutant decreased adhesion area relative to the wild type protein (data not shown). ApFRET analyses revealed that as described earlier (Figure 2), the wild type vinculin interacted with paxillin predominantly in adhesion contacts (Figure 5C; upper panels). In contrast, the interaction between paxillin and the active vinculinT12 mutant was entirely restricted to adhesion contacts (Figure 5C and D). Although plausible, it is unlikely that the T12 mutations directly affect the paxillin/Hic-5 binding domain of vinculin as they are located outside the defined paxillin interaction site [63]. Furthermore, we observed no changes in Pearson's Correlation analysis between paxillin or Hic-5 and the vinculin T12 mutant (data not shown) and unlike talin no difference in paxillin FRAP dynamics were observed in vinculin null cells expressing wild type vinculin or the T12 mutant [71]. No significant FRET (above that of the vinculin880 control level) was observed in adhesions between paxillin and the inactive vinculinA50I (Figure 5D), although a significant increase in FRET efficiency relative to adhesion contact-localized FRET, was observed between paxillin and vinculinA50I at the plasma membrane (Figure 5C and D; 41.5% increase ±6.6, P<0.0005). These analyses indicate that paxillin interacts predominantly with active vinculin in adhesions and vinculin in its inactive conformation at the plasma membrane and therefore suggests that paxillin could play a role in maintaining vinculin in its inactive state at the plasma membrane [68].

Interestingly, a significant increase in Hic-5 FRET efficiency with the active vinculinT12 mutant was observed in adhesions relative to the wild type protein (Figure 5E–G), while no interaction was observed between Hic-5 and the inactive A50I mutant of vinculin either in the adhesion contacts or at the plasma membrane (Figure 5F and G). These data indicate that Hic-5 may interact solely with the active form of vinculin in adhesion contacts (Figure 5E–G) and that this appears to be dependent on the activation of RhoA (Figure 4). Vinculin activation has been shown to peak at the cell periphery during the Rac1-mediated process of cell spreading [68]. However, RhoA activation has also been shown to localize to the leading edge of cell protrusions [51] as well as stimulate vinculin activation and talin binding [72]. Therefore, it is likely that a discrete balance of localized RhoGTPase activity may dictate the levels of paxillin versus Hic-5 binding to vinculin. Alternatively, tension-induced conformational changes in vinculin [73], post-translational modifications (e.g. phosphorylation) of any or all three of the proteins or the absence/presence of auxiliary proteins/phospholipids necessary to maintain complex formation may also influence which interaction predominates. Indeed, it is likely that phosphorylation of paxillin has a role in spatially regulating the interaction between vinculin and paxillin as the latter has been shown to be highly phosphorylated in peripheral focal complexes relative to more mature adhesions [31] and this has been associated with modulating the paxillin-FAK interaction [74]. However, *in vitro* coimmunoprecipitation data suggests that vinculin is able to interact with both phosphorylated and unphosphorylated paxillin [38], [39].

### The interaction between Hic-5 and vinculin predominates in 3D matrix adhesions

Cells rarely encounter 2D substrata during migration *in vivo*, therefore examination of cellular adhesions in 3D ECM model systems is essential for a more complete understanding of the physiologic regulation of cell adhesion and migration. Three dimensional matrix adhesions are morphologically and biochemically distinct from 2D adhesion contacts, with the former containing less phosphorylated FAK [29]. Integrin-mediated adhesions formed in 3D ECM are highly elongated, dynamic, align with fibrous cell-derived ECM (Figure 6A) and contain paxillin, Hic-5 and vinculin [25], [75]. Importantly, the complement of intracellular protein:protein interactions in 3D adhesions remains entirely unexplored.

A key functional/signaling difference between 2D and 3D ECM model systems is the distinct activation profiles of the RhoGTPases. Relative to cell migration on 2D ECM, cells migrating in 3D matrices display reduced Rac1 activation, which promotes uniaxial protrusions and persistent directional migration [75]. Consistent with reduced Rac1 activity in 3D microenvironments we found that despite high colocalization (Figure 6B) the interaction between paxillin and vinculin was attenuated in 3D matrix adhesions (Figure 6C and D).

Importantly, in contrast to 2D (Figure 2F), the highest FRET efficiency was between Hic-5 and vinculin in 3D matrix adhesions (Figure 6C and E). Interestingly, a paxillin and vinculin interaction was still observed at the plasma membrane (Figure 6D) consistent with the effect of reduced Rac1 activity observed in cells in a 2D setting (Figure 3). Importantly, no significant difference between the colocalization of vinculin with paxillin or Hic-5 was observed (Figure 6B). These data indicate that vinculin predominantly interacts with Hic-5 in fibroblast 3D adhesion contacts and are consistent with the reduced levels of Rac1 activity associated with cells migrating in 3D matrices [75] as well as the essential role of Hic-5 in 3D adhesion contact function as observed in breast cancer cells [25].

### Inhibition of Rac1 specifically increases paxillin immobile fraction in adhesions

Adhesion contacts are dynamic structures that must assemble, stabilize and disassemble to enable productive and efficient cell locomotion [24], [25]. The individual proteins that reside in adhesions are also highly dynamic, exhibiting constant exchange with the surrounding plasma membrane and cytoplasm, with proteins remaining in adhesions for seconds to minutes before being replaced [76]. Indeed, the dynamics of individual proteins within an adhesion, have been shown to be intimately linked with adhesion assembly, maturation and disassembly to regulate cell migration [77], [78]. Fluorescence recovery after photobleaching (FRAP) is routinely used to assess the dynamics of individual proteins within adhesions and provides information on the rate at which fluorophore-tagged proteins exchange (half-life, t1/2), as well as identifying the stably associated population of a particular protein within an adhesion (immobile fraction). Having revealed a role for the RhoGTPases in regulating paxillin and Hic-5 interaction with vinculin we used FRAP to examine whether changes in RhoGTPase activity also affects the individual protein's dynamics within adhesions.

Interestingly, pharmacologic inhibition of Rac1 (Figure 7A and B) or expression of the dominant negative N17Rac1 (Figure 7C) resulted in a significant increase in paxillin's immobile fraction (Figure 7B–E) and thus its stability in adhesions. Importantly, this was not dependent on the interaction between paxillin and vinculin in adhesions as this binding is prevented under these conditions (Figure 3). The significant increase in the immobile fraction of paxillin that was observed upon Rac1 inhibition was not seen with vinculin (Figure 7D and E), Hic-5, FAK or zyxin (Figure S6). Furthermore, manipulation of Rac1 activity did not have a significant effect on the t1/2 of either paxillin, Hic-5 or vinculin (Figure 7D, E and Figure S6A, B). The absence of any significant change in t1/2 values suggests that the mechanisms controlling the dynamic, mobile fraction of paxillin are independent of Rac1. Instead, Rac1 activation specifically regulates paxillin retention/stabilization in adhesion contacts.

### Rac1 inhibition increases actopaxin immobile fraction in adhesions and promotes the paxillin-actopaxin interaction

The most stable components of adhesion contacts are likely to be those that perform structural rather than signaling roles. Actopaxin (α-parvin) is a paxillin binding partner, which through its interaction with F-actin may serve a predominantly structural function to regulate cell motility [30], [79]. Therefore, we hypothesized that the interaction between paxillin and actopaxin may be required for the observed Rac1-mediated retention of paxillin in adhesions (Figure 7). Expression of the dominant negative N17Rac1 or treatment with the Rac1 inhibitor resulted in a significant increase in YFP-actopaxin immobile fraction (Figure 8A–C). Furthermore, the interaction between paxillin and actopaxin, as measured by apFRET, was also increased in adhesions upon Rac1 inhibition (Figure 8D and E). Actopaxin has been reported to exist predominantly in a stable complex with ILK and PINCH [80] and this ternary complex has been shown to promote RhoA-dependent adhesion maturation [81]. Interestingly, YFP-ILK FRAP dynamics were unaffected by Rac1 inhibition (Figure S7). Taken together these data suggest that actopaxin and paxillin immobilization in adhesions is regulated by the activity of the RhoGTPases and that their direct interaction may serve a structural role in adhesions under RhoA-mediated tension. Importantly, this also suggests a novel ILK-independent role for actopaxin in RhoGTPase mediated adhesion stabilization.

### Conclusions

The data presented herein enable us to propose a model whereby Rac1 and RhoA activity spatially regulates the direct interaction of vinculin with paxillin and Hic-5 during adhesion maturation. High Rac1 activity as observed at the cell periphery during migration [50], [51], promotes paxillin and active vinculin interaction in newly established focal complexes (Figure 9-1), which is reduced as adhesions mature (Figure 9-2) or lost completely during the transition to high RhoA adhesions (Figure 9-3) as observed in cells under mechanical tension. Indeed, evidence from analysis of adhesion dynamics in paxillin and vinculin knockout fibroblasts suggests that their interaction may be necessary for nascent adhesion contact stabilization and maturation as both cell types exhibit an increase in smaller highly unstable peripheral adhesions [25], [28], [33]. In contrast, the interaction of vinculin with Hic-5 is enhanced later in the adhesion maturation process in conjunction with the activation of RhoA (Figure 9-3) and may thus serve a mechanosensory or mechanotransduction role. Indeed, consistent with a role for Hic-5 in adhesion contact stabilization, fibroblasts devoid of Hic-5 expression exhibit reduced collagen gel contractility due to the inability of mature adhesions to persist and efficiently transmit mechanical force [82].

Given the known roles of both paxillin and Hic-5 as hubs for integrating RhoGTPase signaling [6], [25], it is also plausible that a paxillin/Hic-5-driven feedback loop exists to regulate and fine-tune appropriate spatiotemporal Rac1/RhoA signaling and in turn adhesion dynamics and maturation. For example, paxillin can both activate and inhibit Rac1 through indirect interactions with the Rac1 GEF PIX [83] and the Rac1 GAP CdGAP [84] respectively. Furthermore, paxillin and Hic-5 differentially regulate RhoGTPase activity in cells migrating through 3D microenvironments to modulate cell invasion mechanisms and metastasis [25]. Importantly, despite the profound importance of the RhoGTPases in adhesion contact formation and dynamics in 2D and 3D microenvironments [17], [22], [25], [85], their cellular activation has yet to be localized to sites of adhesion, but rather is tightly restricted at the leading edge of cell protrusions [50], [51]. This may reflect limitations in the spatiotemporal resolution of currently available probes and thus may suggest that adhesion-localized changes in Rac1 and RhoA are small and/or highly transient.

The process of cell migration requires the RhoGTPase-dependent formation and maturation of integrin-mediated adhesion contacts [3]. Our data provide the first description of the molecular consequences of Rac1 and RhoA activation in 2D and 3D adhesions in a cellular environment. Furthermore, these analyses begin to decipher the complexity of protein:protein interactions necessary for adhesion maturation and force transmission during cell migration. The list of proteins that localize to sites of integrin-ECM attachment is ever expanding [4], yet the complex interrelationship between these proteins is largely undetermined. FRET and FRAP approaches in combination with biochemical analyses, bioinformatics as well as super-resolution imaging techniques, such as iPALM [86], will enable modeling of adhesion ultrastructure and dynamics at a molecular level during physiologic and pathophysiologic cell migration.

## Materials and Methods

### Antibodies and reagents

To specifically identify endogenous paxillin and Hic-5 by Western blotting and immunofluorescence the mouse anti-paxillin (clone 165) and mouse anti-Hic-5 (BD Biosciences, Franklin Lakes, NJ) were utilized. For detection of both paxillin and Hic-5 by immunofluorescence mouse anti-paxillin (clone 349) was used (BD Biosciences). Analysis of endogenous and YFP-tagged vinculin and vinculin mutants by immunofluorescence and Western blotting as stated was performed using the mouse anti-vinculin (VIN-11-5) (Sigma Aldrich, St Louis, MO) and rabbit anti-GFP (Santa Cruz Biotechnology, Santa Cruz, CA). Other antibodies used for immunofluorescence and Western blotting include mouse anti-c-myc (clone 9E10; Developmental Studies Hybridoma Bank at the University of Iowa, Iowa City, IA); goat anti-transaldolase (Santa Cruz Biotechnology); mouse anti-β1 integrin (BD Biosciences); mouse anti-alpha tubulin, rabbit anti-calnexin and rabbit anti-fibronectin (Sigma Aldrich); rabbit anti-phospho-myosin light chain 2 (Ser19) (Cell Signaling Technology, Inc., Danvers, MA),. Rhodamine-conjugated phalloidin (Invitrogen, Carlsbad, CA) and 4′,6-diamidino-2-phenylindole (DAPI) (Sigma Aldrich) were used for fluorescent detection of F-actin and the cell nucleus respectively. The Rac1 inhibitor, NSC23766, was used at a concentration of 50 µM and was purchased from EMD Chemicals (San Diego, CA).

### Cell culture

NIH 3T3 (ATCC, Manassas, VA) cells were cultured in Dulbecco's modified Eagle's medium (DMEM) supplemented with 10% (v/v) fetal calf serum (Invitrogen), L-glutamine, 1 mM sodium pyruvate and 1% (v/v) penicillin and streptomycin. Transient transfection of tagged proteins was performed using Lipofectamine LTX and Optimem I (Invitrogen) medium using 4µg cDNA unless otherwise stated.

### Immunofluorescence

Immunofluorescence was performed as previously described [25]. Briefly, 4×10^4^ NIH 3T3 cells were spread on 10 µg/ml FN or 3D cell-derived matrices (CDM)-coated coverslips for indicated times in complete media then washed once with PBS. Cells were then fixed for 15 minutes at room temperature with 4% paraformaldehyde containing 1% Triton® X-100 diluted in PBS. Samples were then washed three times with PBS and paraformaldehyde quenched at room temperature with 0.1 M glycine in PBS for 15 mins. Samples were then washed with PBS and incubated with 3% (w/v) bovine serum albumin (BSA) diluted in PBS overnight at 4°C prior to immunofluorescence staining. Fixed cells were stained with primary antibodies as indicated in 3% (w/v) BSA. PBS with 0.05%Tween-20 was used for subsequent washes. Cells were imaged using the Leica SP5 scanning confocal with a HCX PL APO 63x/1.40–0.60 OIL λ BL objective (Leica, Bannockburn, IL).

### Acceptor photobleaching Fluorescence Resonance Energy Transfer (apFRET)

NIH 3T3 cells were transfected using Lipofectamine LTX following the manufacturers standard instructions with a cDNA ratio of 2∶1 donor to acceptor. Twenty-four hours post-transfection, 35-mm glass-bottomed, poly-L-lysine dishes (Mat-tek) were coated with 10 μg/ml FN diluted in PBS containing calcium and magnesium overnight at 4°C. To prevent non-specific cell binding, the dishes were blocked with 10 mg/ml heat-denatured bovine serum albumin (BSA) for 30 minutes. 4×10^4^ cells were added to each well and allowed to attach and spread overnight 37°C, 5% (v/v) CO_2_. Remaining cells were lysed in hot 1x reducing sample buffer for protein expression Western blot analyses. Where stated, cells were treated with vehicle (dH_2_0) or 50 µM Rac1 inhibitor 4 hours prior to fixation. The spread cells were then fixed for 15 minutes at room temperature with 4% (w/v) paraformaldehyde with 1% Triton® X-100 diluted in PBS to deplete the cytosolic fraction. The dishes were then washed with PBS and the paraformaldehyde quenched with 0.1 M glycine in PBS for 15 minutes. Fixed cells were imaged in the YFP and RFP channel on a Leica AF6000 LX deconvolution microscope using a 100x/1.40–0.70 HCX PL APO objective and Leica DFC350 FX camera at 2×2 binning. FRET imaging and calculations were performed as described previously [42]. Briefly, a YFP image (donor) was captured before and after photobleaching the mRFP (acceptor) in the RFP channel. Following background subtraction, the percentage FRET efficiency was calculated on a pixel by pixel basis, as 100×[1–(donor intensity before photobleaching/donor intensity after photobleaching)], using ImageJ software (Rasband, W.S., National Institutes of Health, Bethesda, MD, USA; http://rsb.info.nih.gov/ij/). FRET images were smoothed and displayed as a color intensity scale. If the donor (YFP) and acceptor (mRFP) are within FRET proximity (<10 nm), then upon donor excitation, energy is transferred to the acceptor causing donor quenching and acceptor excitation. Thus, if FRET proximity is achieved, an increase in donor emission will be observed upon acceptor photobleaching. [40], [41], [42].

To control for pixel shift aberrations and edge artifacts due to fluctuations in temperature and focus drift during mRFP photobleaching, the YFP images taken before and after photobleaching were merged and pixel alignment fidelity assessed and corrected. Only images in which all pixels could be aligned as determined by multiple line profile through adhesion contacts and Pearson's Correlation coefficient analyses were used for subsequent FRET calculations. To measure FRET within adhesion contacts, a mask was created from a thresholded YFP image before photobleaching and measurements restricted to adhesions of defined area. Membrane FRET was calculated by averaging the FRET efficiency in all non-adhesion contact areas. Pearson's correlation, line profile and adhesion area analyses were performed using the Image J software. Pearson's correlation coefficients were calculated from YFP and mRFP channel before photobleaching images after background subtraction using the Image Correlator Plus plug-in. The Pearson's correlation reflects the linear relationship between the localized intensities of the two fluorophore-tagged proteins.

### Cell fractionation

NIH 3T3 cells were spread overnight in the presence of serum on two heat-denatured BSA blocked, 10 μg/ml FN-coated 10 cm dishes at 4×10^5^ cells/dish. Cells were then treated with either vehicle or 50 μM Rac1 inhibitor 4 hours prior to cell fractionation. Cells were then fractionated using the Qproteome ® cell compartment kit (Qiagen) following the manufacturer's instructions. The lysates collected were then analyzed by Western blotting for enrichment of cell compartment proteins, transaldolase (cytosol), β1 integrin and calnexin (membrane) to confirm fractionation fidelity.

### Coimmunoprecipitation

Global intracellular association of endogenous and exogenous fluorophore-tagged paxillin or Hic-5 with vinculin was assessed using coimmunoprecipitation. 1×10^5^ NIH 3T3 cells (transfected as stated using Lipofectamine LTX; *see Experimental Procedures, apFRET*) were spread on heat-denatured BSA blocked 10 µg/ml FN-coated 60 mm dishes overnight at 37°C and 5% CO_2_ in the presence of serum. Cells were treated with vehicle or 50 µM Rac1 inhibitor 4 hours before being resuspended in lysis buffer (50 mM Tris-HCl, pH 7.6, 10% (v/v) glycerol, 100 mM NaCl, 1 mM EDTA, 0.75% (v/v) Triton® X-100, 2 mM NaVO_3_, 1 mM PMSF and 10 µg/ml leupeptin). Lysates were incubated on ice for 10 minutes and spun at 18,000 g for 5 minutes. The supernatants were then collected and incubated with either 5 µg mouse anti-paxillin clone165 (BD) or rabbit anti-GFP (Santa Cruz) for 2 hours rotating at 4°C followed by a 1 hour incubation with protein A/G beads (Santa Cruz). Samples were spun at 3,800 g for 1 minute and washed three times with lysis buffer and bound protein assessed by Western blotting.

### 3D cell-derived matrix (CDM) generation

Cell-derived matrices were generated as previously described [25] using primary human foreskin fibroblasts (ATCC).

### Fluorescence Recovery After Photobleaching (FRAP)

A Leica TCS SP5 laser-scanning confocal microscope equipped with a multi-line 100 mW Argon laser as well as a 1mW HeNe 543 nm lasers was used for all FRAP experiments. NIH 3T3 cells were transfected with cDNAs as stated using Lipofectamine LTX. Twenty-four hours post-transfection cells were plated on pre-blocked FN-coated glass-bottomed dishes in the presence of serum for 16 hours. Where stated, cells were treated with vehicle or 50 µM Rac1 inhibitor 4 hours prior to FRAP analyses. Cells expressing the YFP- or GFP-tagged constructs were imaged using a HCX PL APO 63x/1,30 Glyc Corr 37°C objective in an environmentally controlled chamber at 37°C and 5% CO_2_ in complete DMEM growth medium.

Initial fluorescence intensity was measured at low laser power (12%) followed by photobleaching of individual adhesion contacts >1 µm^2^ away from the cell periphery. YFP-tagged proteins were photobleached using the 496, 514 and 543 nm laser lines while GFP-tagged proteins were photobleached using the 476, 488 and 496 nm laser lines at 100% laser power for 4 iterations. Fluorescence recovery was then monitored at low laser power for 80–120 secs with images acquired at 2 sec intervals (40 to 60 iterations). Images were background subtracted and corrected for photobleaching as determined through monitoring fluorescence intensity of a non-photobleached adhesion contact in the image over the recovery period. FRAP analyses were performed using Image J software. The percentage mobile (M*_f_*) and immobile fractions (I*_f_*) were determined by comparing the fluorescence intensity in the photobleached region after recovery to plateau (F_∞_) with the intensity before (F*_i_*) and initially after bleaching (F_0_) using the equations M*_f_*  = 100×(F_∞_– F_0_)/(F*_i_* – F_0_) and I*_f_*  = 100– M*_f_*. The t1/2 of recovery was determined from the kinetic plot of fluorescence recovery and calculated using GraphPad Prism software. A minimum of 10 adhesion contacts from 5 individual cells, were photobleached and fluorescence recovery monitored per tagged protein and condition (n = 3). Therefore, recovery curves represent data from a minimum of 30 individual adhesions from 3 separate experiments. To avoid misinterpretations and bias arising from proteins exhibiting varying dynamics depending on their specific location within an individual adhesion contact as previously reported [87], [88], entire adhesions were photobleached and their fluorescence recovery monitored.

### Statistical Analyses

Independent data sets were determined to exhibit a normal distribution using the Shapiro-Wick and Kolmogorov-Smirnov tests of normality and were subjected to an unpaired Student's t test. Statistical analyses were performed using Excel and GraphPad Prism software.

## Supporting Information

Figure S1 **Fluorescently tagged paxillin and vinculin used for FRET experiments are expressed at endogenous levels and interact in all adhesion areas.** (A) Western blots of NIH 3T3 cell lysates of cells transfected with YFP-tagged donor and mRFP-tagged acceptors indicating similar expression levels to the endogenous proteins. (B) Merged overlay of the vinculin-YFP raw images before (red) and after (green) mRFP-paxillin photobleaching. Line profiles indicate pixel colocalization (no pixel shift aberrations), as well as FRET which is seen as an increase in the fluorescence intensity post mRFP-paxillin photobleaching (green line).
(TIF)

Figure S2 **Fluorescently tagged Hic-5 and vinculin are expressed at endogenous levels and FRET in all adhesion areas above control levels.** (A) Western blots of NIH 3T3 cell lysates of cells transfected with fluorophore-tagged donor and acceptor as indicated showing similar expression levels to the endogenous proteins. (B) Merged overlay of the vinculin-YFP raw images before (red) and after (green) mRFP-Hic-5 photobleaching. Line profiles indicate pixel colocalization, as well as FRET which is seen as an increase in the fluorescence intensity post mRFP-Hic-5 photobleaching (green line). Raw and processed FRET images of control (C) mRFP and vinculin-YFP and (D) mRFP-paxillin and zyxin-YFP FRET pairs. Line profiles indicate no increase in YFP fluorescence after acceptor photobleaching and thus no significant FRET.
(TIF)

Figure S3 **Expression of N17Rac1 and V14RhoA enhances myosin light chain 2** (**MLC2**) **phosphorylation.** (A) Representative Western blot and (B) quantitation of MLC2 phosphorylation (p-MLC2; Ser19) indicative of elevated RhoA signaling in cells expressing the dominant negative (N17) Rac1 and dominant active (V14) RhoA. N = 4 individual experiments and * = P<0.05. (C) Representative Western blot and (D) quantitation of MLC2 phosphorylation in cells ±50 μM Rac1 inhibitor. N = 3 and *** = p<0.0005.
(TIF)

Figure S4 **Pearson's Correlation analyses reveals that the changes of FRET are not due to changes in colocalization.** Pearson's Correlation analyses of cells used for FRET quantitation expressing either mRFP-paxillin and vinculin-YFP or mRFP-Hic-5 and vinculin-YFP with (A and C) Rac1 mutant constructs and (B and D) ±50 μM Rac1 inhibitor. No significant difference in colocalization was ever observed.
(TIF)

Figure S5 **Inhibition of Rac1 or activation of RhoA promotes increased FRET between paxillin and vinculin in the cytosol/membrane relative to the adhesion contacts.** Quantitation of the relative FRET efficiency between mRFP-paxillin and vinculin-YFP in all adhesion contacts in the cell versus the surrounding cytosol/membrane upon expression of (A) wtRac1 or N17Rac1 and (B) ±50 μM Rac1 inhibitor treatment. Data represents a minimum of n = 3 individual experiments and 7 individual cells. *  = P<0.05 and ** = P<0.005.
(TIF)

Figure S6 **Inhibition of Rac1 has no effect on Hic-5, FAK or zyxin FRAP dynamics.** FRAP recovery curves and immobile fraction data for adhesions of cells expressing (A) GFP-Hic-5±50 μM Rac1 inhibitor, (B) GFP-Hic-5 (C) YFP-FAK and (D) zyxin-YFP with wild type or dominant negative N17Rac1. Data are combined analyses from a minimum of 10 adhesions from 5 cells and 3 individual experiments. No significant differences in either t1/2 or immobile fraction were observed upon manipulation of Rac1 activity.
(TIF)

Figure S7 **Inhibition of Rac1 has no effect on ILK FRAP dynamics.** FRAP recovery curves and immobile fraction data for adhesions of cells expressing YFP-ILK with wild type or dominant negative N17Rac1. Data are combined analyses from a minimum of 10 adhesions from 5 cells and 3 individual experiments. No significant differences in either t1/2 or immobile fraction were observed upon manipulation of Rac1 activity.
(TIF)
