# Supplementary figures and images for: Paxillin and Hic-5 Interaction with Vinculin Is Differentially Regulated by Rac1 and RhoA
Source: PLoS One. 2012 May 22;7(5):e37990. doi: 10.1371/journal.pone.0037990 (PMC3358283; doi:10.1371/journal.pone.0037990)

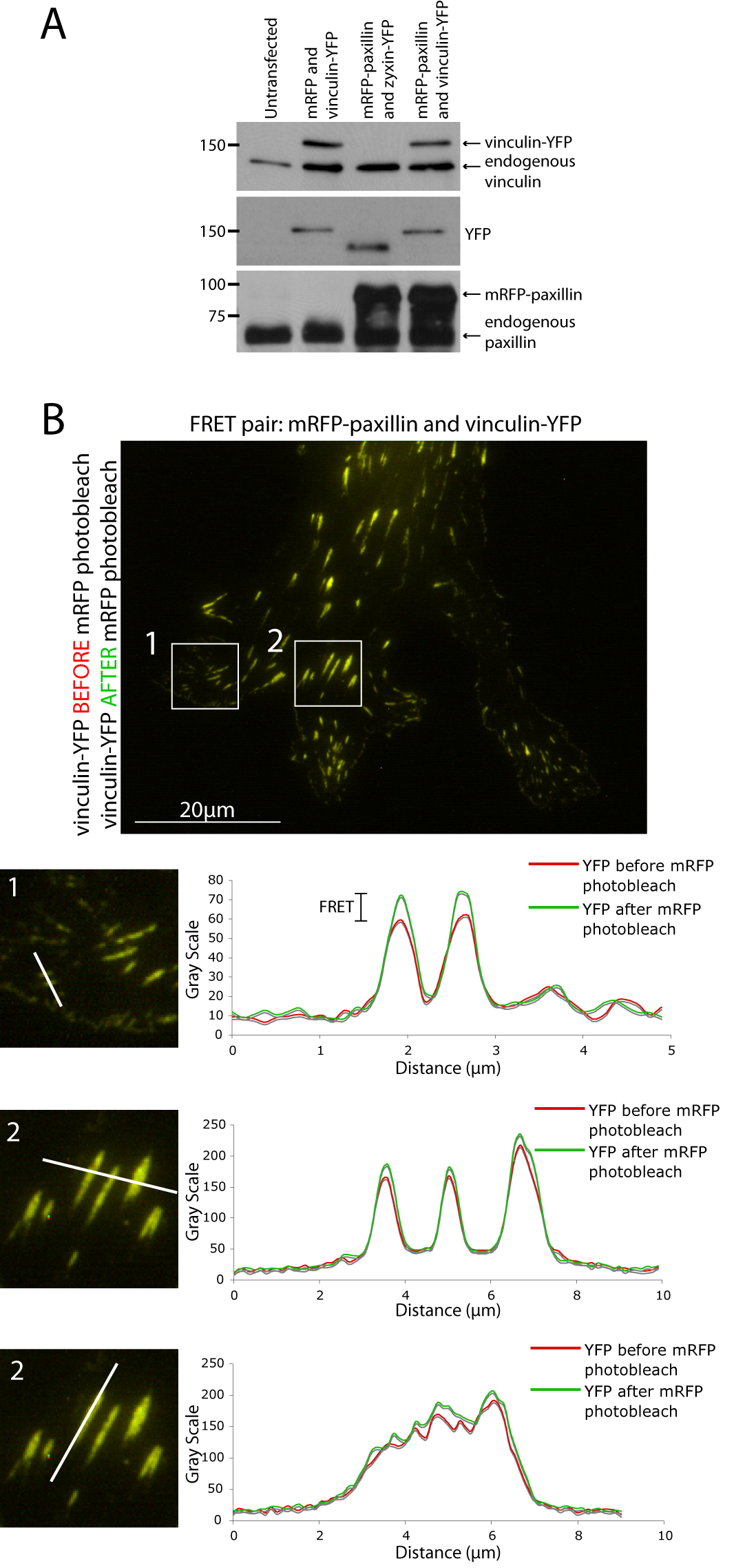

Supplement: Figure S1 — Fluorescently tagged paxillin and vinculin used for FRET experiments are expressed at endogenous levels and interact in all adhesion areas. (A) Western blots of NIH 3T3 cell lysates of cells transfected with YFP-tagged donor and mRFP-tagged acceptors indicating similar expression levels to the endogenous proteins. (B) Merged overlay of the vinculin-YFP raw images before (red) and after (green) mRFP-paxillin photobleaching. Line profiles indicate pixel colocalization (no pixel shift aberrations), as well as FRET which is seen as an increase in the fluorescence intensity post mRFP-paxillin photobleaching (green line). (TIF) [file pone.0037990.s001.tif]

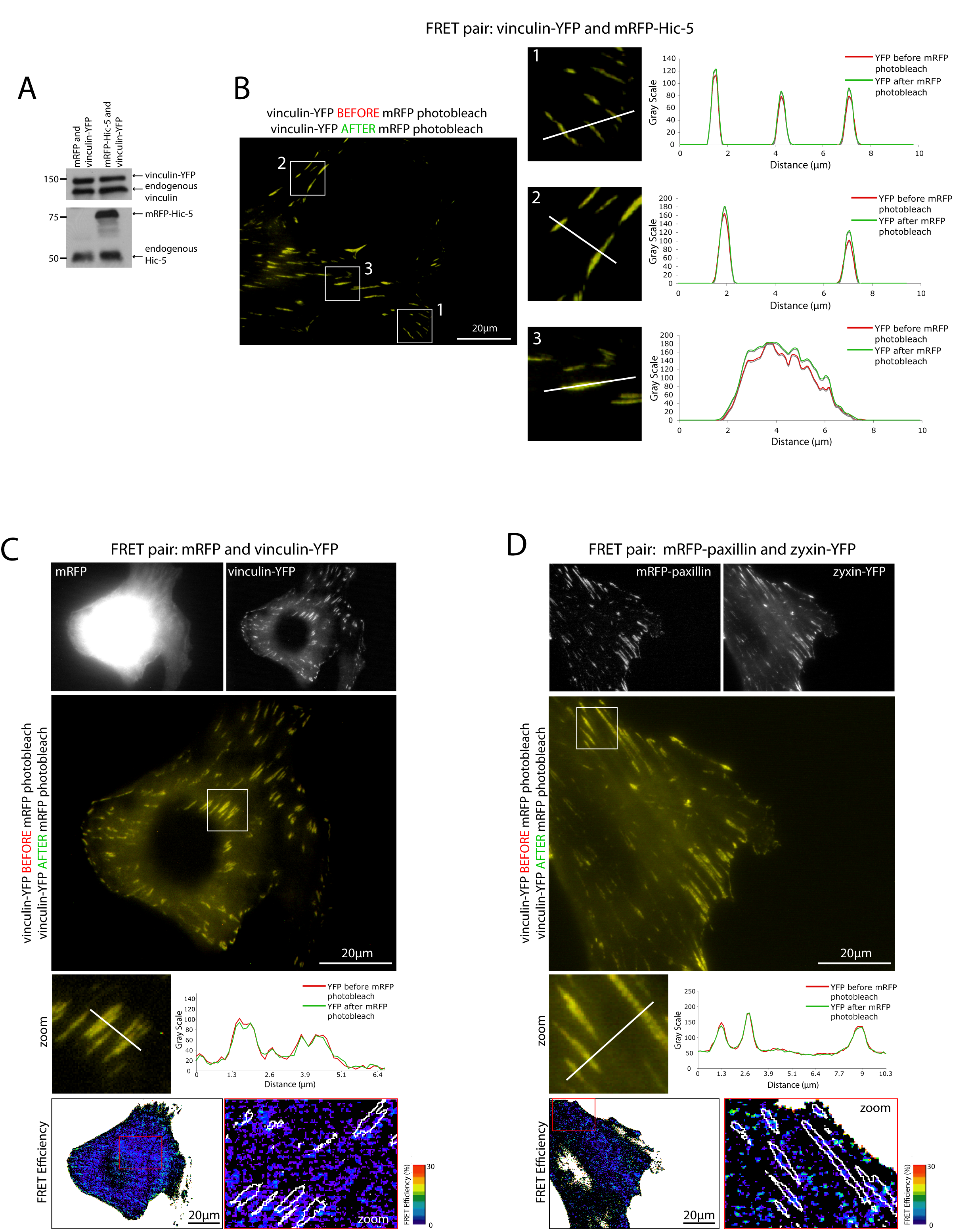

Supplement: Figure S2 — Fluorescently tagged Hic-5 and vinculin are expressed at endogenous levels and FRET in all adhesion areas above control levels. (A) Western blots of NIH 3T3 cell lysates of cells transfected with fluorophore-tagged donor and acceptor as indicated showing similar expression levels to the endogenous proteins. (B) Merged overlay of the vinculin-YFP raw images before (red) and after (green) mRFP-Hic-5 photobleaching. Line profiles indicate pixel colocalization, as well as FRET which is seen as an increase in the fluorescence intensity post mRFP-Hic-5 photobleaching (green line). Raw and processed FRET images of control (C) mRFP and vinculin-YFP and (D) mRFP-paxillin and zyxin-YFP FRET pairs. Line profiles indicate no increase in YFP fluorescence after acceptor photobleaching and thus no significant FRET. (TIF) [file pone.0037990.s002.tif]

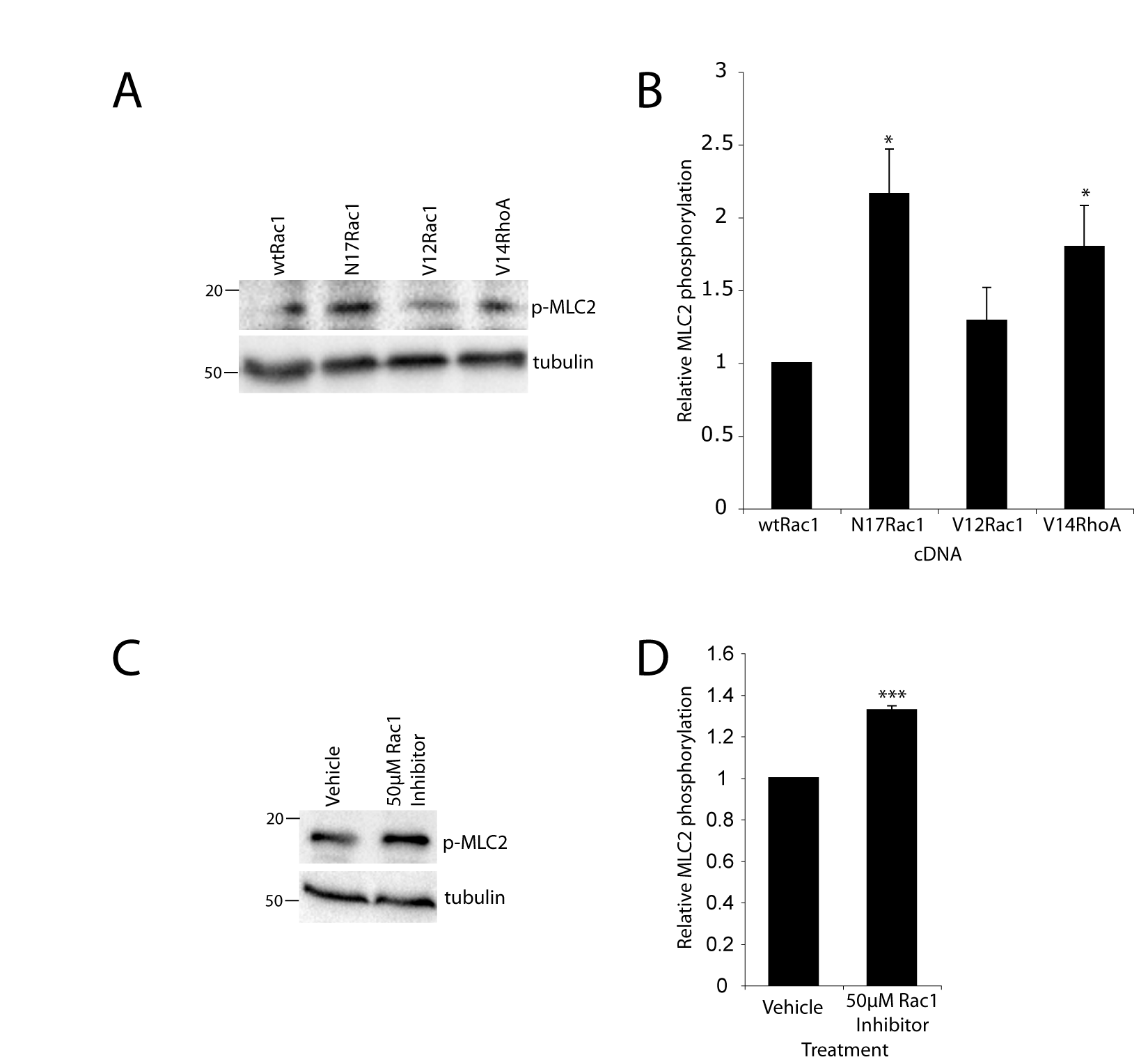

Supplement: Figure S3 — Expression of N17Rac1 and V14RhoA enhances myosin light chain 2 (MLC2) phosphorylation. (A) Representative Western blot and (B) quantitation of MLC2 phosphorylation (p-MLC2; Ser19) indicative of elevated RhoA signaling in cells expressing the dominant negative (N17) Rac1 and dominant active (V14) RhoA. N = 4 individual experiments and * = P<0.05. (C) Representative Western blot and (D) quantitation of MLC2 phosphorylation in cells ±50 μM Rac1 inhibitor. N = 3 and *** = p<0.0005. (TIF) [file pone.0037990.s003.tif]

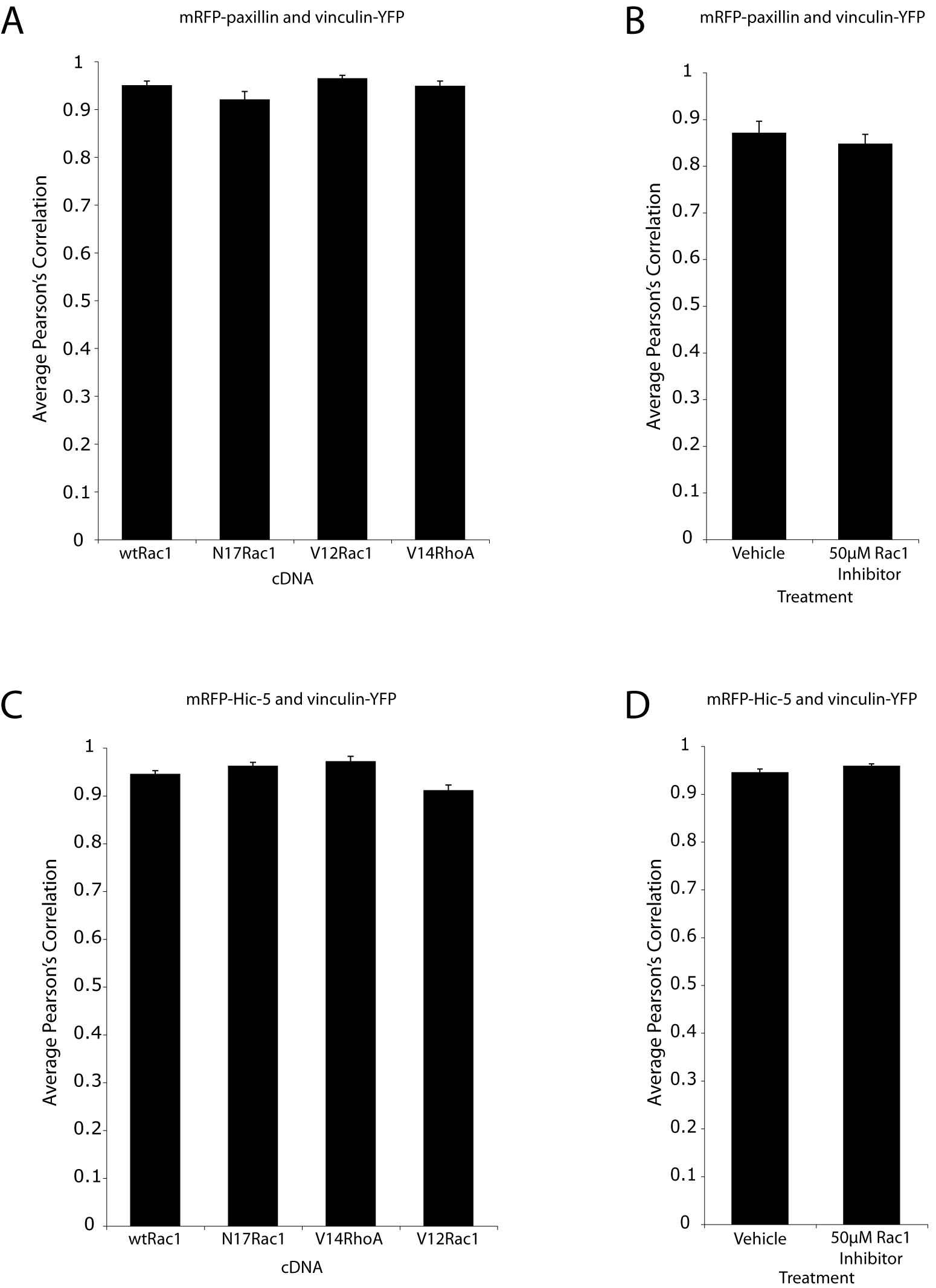

Supplement: Figure S4 — Pearson's Correlation analyses reveals that the changes of FRET are not due to changes in colocalization. Pearson's Correlation analyses of cells used for FRET quantitation expressing either mRFP-paxillin and vinculin-YFP or mRFP-Hic-5 and vinculin-YFP with (A and C) Rac1 mutant constructs and (B and D) ±50 μM Rac1 inhibitor. No significant difference in colocalization was ever observed. (TIF) [file pone.0037990.s004.tif]

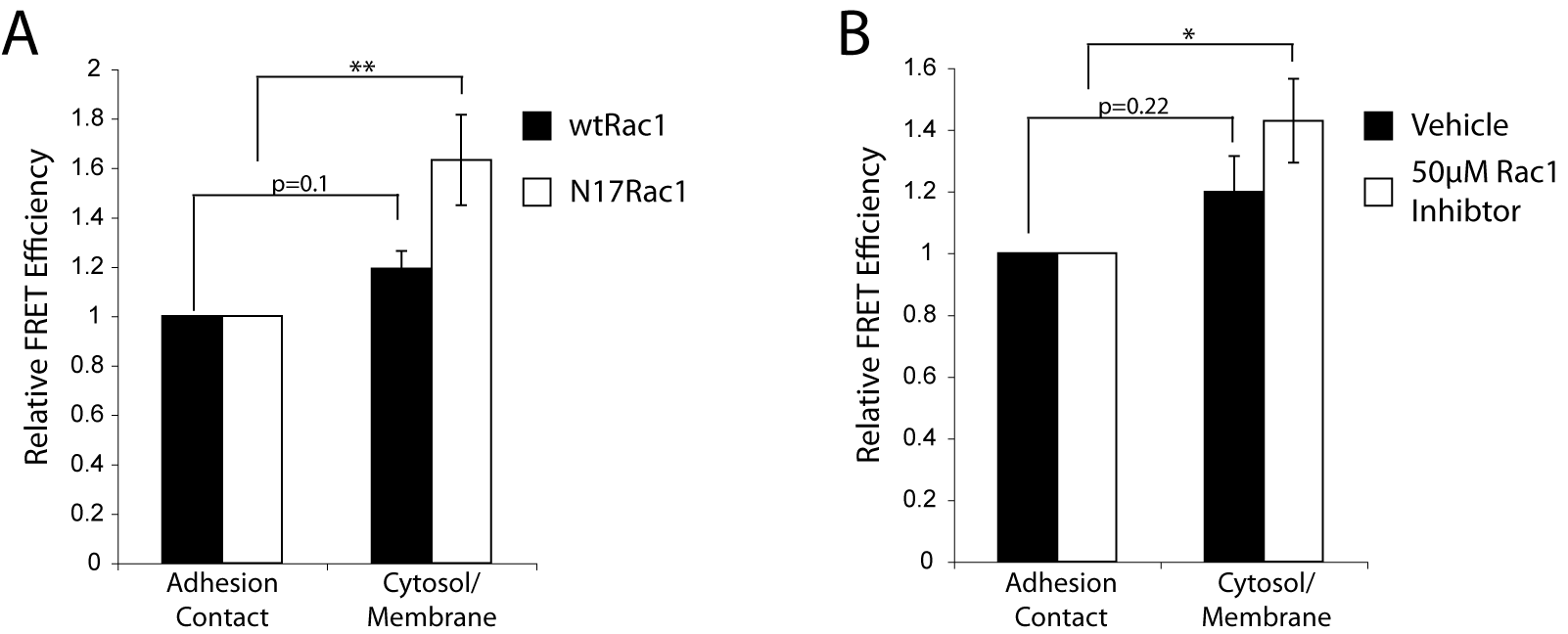

Supplement: Figure S5 — Inhibition of Rac1 or activation of RhoA promotes increased FRET between paxillin and vinculin in the cytosol/membrane relative to the adhesion contacts. Quantitation of the relative FRET efficiency between mRFP-paxillin and vinculin-YFP in all adhesion contacts in the cell versus the surrounding cytosol/membrane upon expression of (A) wtRac1 or N17Rac1 and (B) ±50 μM Rac1 inhibitor treatment. Data represents a minimum of n = 3 individual experiments and 7 individual cells. * = P<0.05 and ** = P<0.005. (TIF) [file pone.0037990.s005.tif]

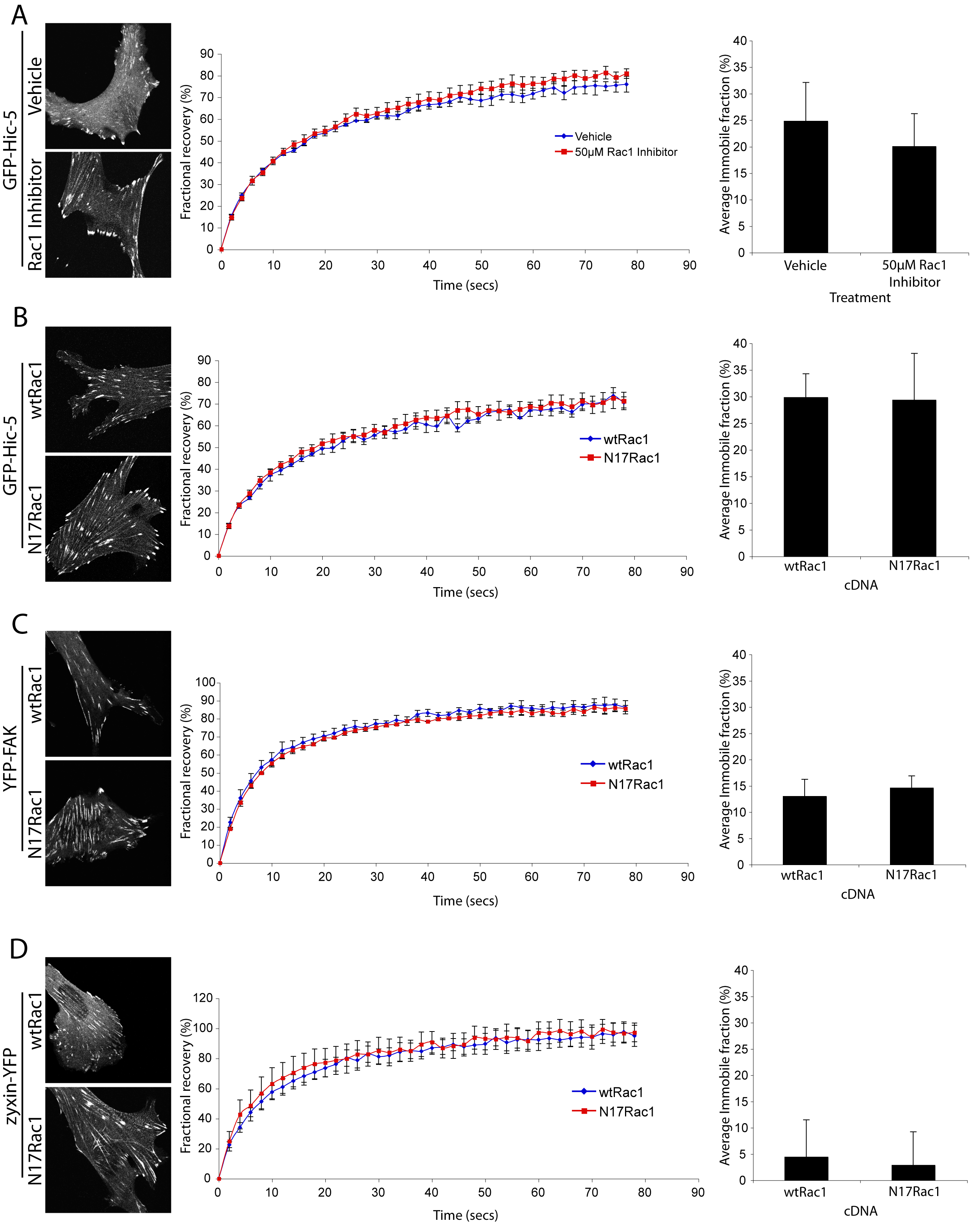

Supplement: Figure S6 — Inhibition of Rac1 has no effect on Hic-5, FAK or zyxin FRAP dynamics. FRAP recovery curves and immobile fraction data for adhesions of cells expressing (A) GFP-Hic-5±50 μM Rac1 inhibitor, (B) GFP-Hic-5 (C) YFP-FAK and (D) zyxin-YFP with wild type or dominant negative N17Rac1. Data are combined analyses from a minimum of 10 adhesions from 5 cells and 3 individual experiments. No significant differences in either t1/2 or immobile fraction were observed upon manipulation of Rac1 activity. (TIF) [file pone.0037990.s006.tif]

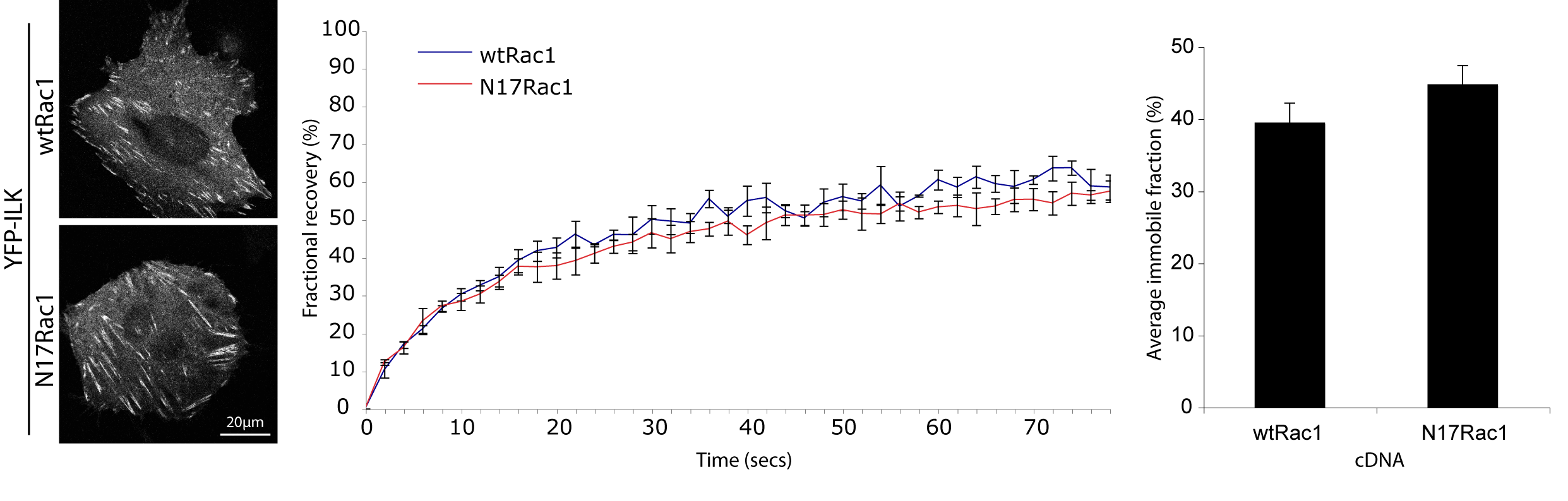

Supplement: Figure S7 — Inhibition of Rac1 has no effect on ILK FRAP dynamics. FRAP recovery curves and immobile fraction data for adhesions of cells expressing YFP-ILK with wild type or dominant negative N17Rac1. Data are combined analyses from a minimum of 10 adhesions from 5 cells and 3 individual experiments. No significant differences in either t1/2 or immobile fraction were observed upon manipulation of Rac1 activity. (TIF) [file pone.0037990.s007.tif]
